# Unraveling the role of MADS transcription factor complexes in apple tree dormancy

**DOI:** 10.1111/nph.17710

**Published:** 2021-09-23

**Authors:** Vítor da Silveira Falavigna, Edouard Severing, Xuelei Lai, Joan Estevan, Isabelle Farrera, Véronique Hugouvieux, Luís Fernando Revers, Chloe Zubieta, George Coupland, Evelyne Costes, Fernando Andrés

**Affiliations:** ^1^ UMR AGAP Institut Univ Montpellier CIRAD INRAE Institut Agro F‐34398 Montpellier France; ^2^ Department of Plant Developmental Biology Max Planck Institute for Plant Breeding Research 50829 Cologne Germany; ^3^ Laboratoire de Physiologie Cellulaire et Végétale Université Grenoble‐Alpes CNRS CEA INRAE IRIG‐DBSCI 38000 Grenoble France; ^4^ Embrapa Uva e Vinho Bento Gonçalves RS 95701‐008 Brazil

**Keywords:** apple tree (*Malus domestica*), bud dormancy, budbreak, climate change, *DAM* genes, seq‐DAP‐seq, *SVP* genes

## Abstract

- A group of MADS transcription factors (TFs) are believed to control temperature‐mediated bud dormancy. These TFs, called DORMANCY‐ASSOCIATED MADS‐BOX (DAM), are encoded by genes similar to *SHORT VEGETATIVE PHASE* (*SVP*) from Arabidopsis. MADS proteins form transcriptional complexes whose combinatory composition defines their molecular function. However, how MADS multimeric complexes control the dormancy cycle in trees is unclear.
- Apple MdDAM and other dormancy‐related MADS proteins form complexes with MdSVPa, which is essential for the ability of transcriptional complexes to bind to DNA. Sequential DNA‐affinity purification sequencing (seq‐DAP‐seq) was performed to identify the genome‐wide binding sites of apple MADS TF complexes. Target genes associated with the binding sites were identified by combining seq‐DAP‐seq data with transcriptomics datasets obtained using a glucocorticoid receptor fusion system, and RNA‐seq data related to apple dormancy.
- We describe a gene regulatory network (GRN) formed by MdSVPa‐containing complexes, which regulate the dormancy cycle in response to environmental cues and hormonal signaling pathways. Additionally, novel molecular evidence regarding the evolutionary functional segregation between DAM and SVP proteins in the Rosaceae is presented.
- MdSVPa sequentially forms complexes with the MADS TFs that predominate at each dormancy phase, altering its DNA‐binding specificity and, therefore, the transcriptional regulation of its target genes.

## Introduction

Temperate trees adjust their growth and flowering cycles to seasonal environmental conditions. This plasticity is conferred by sensing mechanisms and signaling pathways that reprogram a population of pluripotent cells called meristems. Photoperiodic reduction and low temperatures in winter serve as signals to induce growth cessation, bud formation, and a meristematic state of rest called endodormancy, during which visible growth is inhibited. Endodormant buds can recover their growth competence after exposure to a certain period of low temperatures (Lang *et al*., 1987; Anderson, 2015); this ability is species‐ and cultivar‐dependent and is known as chilling requirement (CR). Chilling requirement fulfillment leads to a vast genetic reprogramming of the shoot apical meristem (SAM) (Ruttink *et al*., 2007; Takeuchi *et al*., 2018; Vimont *et al*., 2019; Moser *et al*., 2020), which undergoes an ecodormant phase, that is, the ability to resume growth after exposure to sufficient warm temperatures. In springtime, budbreak and flowering take place when climatic conditions are favorable.

Despite the importance of a well‐adjusted dormancy cycle for flowering timing and fruit production, our understanding of how this process is controlled in the Rosaceae and other significant crops is still in its infancy. A group of MADS transcription factors (TF) are major regulators of the dormancy cycle in many Rosaceous fruit trees. These genes are referred as *DORMANCY‐ASSOCIATED MADS‐BOX* (*DAM*), and they were first identified through their genetic association with the nondormant phenotype of the *evergrowing* mutant of peach (*Prunus persica*) (Bielenberg *et al*., 2008). *DAM* genes belong to the classical MIKC type of MADS TF and are similar to *SHORT VEGETATIVE PHASE* (*SVP*) from Arabidopsis (Jiménez *et al*., 2009). Further genetic studies have identified *DAM* genes within quantitative trait loci related to the dormancy cycle in many Rosaceous species (Castède *et al*., 2015; Allard *et al*., 2016; Gabay *et al*., 2017; Li *et al*., 2019). Notably, the expression of the *DAM* genes displays distinct seasonal patterns (Falavigna *et al*., 2019). A subgroup of them is induced by low temperatures and shows maximal expression in the middle of the winter, which has been associated with the physiological establishment and maintenance of endodormancy (Sasaki *et al*., 2011; Falavigna *et al*., 2019). Characteristically, these genes are progressively downregulated after prolonged exposure to cold, reaching minimal levels of expression upon CR fulfillment and endodormancy release (Falavigna *et al*., 2019; Vimont *et al*., 2019). Ectopic expression of the Japanese apricot (*Prunus mume*) *PmDAM6* gene in poplar induced growth cessation, bud set and bud endodormancy (Sasaki *et al*., 2011). *MdDAM1* gene silencing in transgenic apple trees (*Malus domestica*) led to a nondormant and constant growing phenotype (Moser *et al*., 2020), in a similar fashion to the simultaneous downregulation of three *MdDAM* genes (*MdDAM1*, *MdDAM4* and *MdDAMb*) and two *MdSVP* genes (*MdSVPa* and *MdSVPb*) (Wu *et al*., 2021). Conversely, *MdDAMb* and *MdSVPa* overexpression caused growth inhibition and delayed budbreak but did not affect growth cessation or endodormancy induction (Wu *et al*., 2017). The role of *SVP* ortholog genes (*SVP*‐like) in repressing budbreak has been further demonstrated in hybrid aspen (Singh *et al*., 2018). The functional characterization of *DAM*‐ and *SVP*‐like genes suggests the existence of one or more temperature‐mediated gene regulatory networks (GRNs) controlling dormancy in which these genes play a central role. Clearly defining these GRNs is essential to obtaining a better understanding of how budbreak and flowering is temporally modulated in fruit trees.

In Arabidopsis, SVP inhibits floral transition by affecting GRNs that integrate environmental cues and endogenous hormonal signals (Gregis *et al*., 2013). Similarly to other MADS TFs, SVP forms dimers or tetramers that bind to specific DNA sequences termed CArG‐boxes (CC(A/T)_6_GG) (Davies *et al*., 1996; Egea‐Cortines *et al*., 1999). The oligomeric composition of these complexes determines the genomic regions to which they bind, allowing multiple transcriptional responses via combinations of distinct TFs (Lai *et al*., 2019). For example, FLOWERING LOCUS C (FLC), a MADS TF that represses flowering until the plant is exposed to a prolonged period of low temperatures in a process known as vernalization (Michaels & Amasino, 1999), is a binding partner of SVP. SVP and FLC form a transcriptional repressive complex that inhibits floral transition (Li *et al*., 2008). The molecular mechanism underlying the control of the dormancy cycle by temperature in trees is believed to be analogous to that described for the vernalization pathway in Arabidopsis (Leida *et al*., 2012; Falavigna *et al*., 2019). This notion is supported by two main pieces of evidence: the cold‐mediated transcriptional regulation of *DAM* genes, which resembles that of *FLC* in Arabidopsis, and by the similar role of *SVP*‐like genes in repressing budbreak date and flowering time in fruit trees and Arabidopsis, respectively (Hartmann *et al*., 2000; Wu *et al*., 2017; Singh *et al*., 2018). Furthermore, the function of FLC‐like TFs has been recently related to tree dormancy control (Porto *et al*., 2015; Urrestarazu *et al*., 2017; Miotto *et al*., 2019). However, there is no proof yet of the existence of multimeric complexes formed by MADS TFs that govern GRNs controlling the dormancy cycle in trees. Here, we show that apple DAM‐, FLC‐ and SVP‐like complexes operate in GRNs that integrate environmental and hormonal signaling pathways to regulate dormancy. Moreover, we propose the existence of an evolutionary functional segregation between DAM and SVP‐like TFs. Whereas SVP flowering‐related functions are conserved between phylogenetically distant species, DAM TFs have diverged to play specific dormancy roles in Rosaceous tree species.

## Materials and Methods

### Plant material

Three dormant buds from ‘Royal Gala’ (*Malus domestica* Borkh) trees were harvested per plant (three sampling blocks of four plants each) at eight timepoints. Samples were immediately frozen in liquid nitrogen. The dormant stage of the buds was evaluated under forcing conditions (16 h : 8 h, light : dark photoperiod at 22°C) using Tabuenca’s test (Methods S1) (Tabuenca, 1964).

### Gene expression studies

Total RNA was isolated using the Spectrum Plant Total RNA kit (Sigma‐Aldrich), and the SuperScript III First‐Strand Synthesis System (Thermo Fisher Scientific, Waltham, MA, USA) was used for cDNA synthesis. Real‐time polymerase chain reaction was performed using the LightCycler 480 instrument (Roche, Mannheim, Germany) as described previously (Falavigna *et al*., 2018). The primers used are listed in Table S1. *MdMDH* and *MdWD40* were used as reference genes (Perini *et al*., 2014).

### *pENTR* vector construction

*MdDAM1*, *MdDAM2*, *MdDAM4*, *MdDAMb*, *MdSVPa*, *MdSVPb*, *MdFLC*, *SVP* and *Venus* were cloned into *pDONR201* and *pDONR207* vectors (Karimi *et al*., 2007) using the Gateway system (Thermo Fisher Scientific). *MdDAM1*, *MdDAM4*, *MdFLC* and *MdSVPa* without the stop codon were cloned into *pDONR207*, and recombined into the *pBEACON‐GR* vector (kindly provided by Dr Gloria Coruzzi) using the Gateway system, and genes fused to the glucocorticoid receptor (GR) were amplified and cloned into the *pDONR207* vector. Truncated versions retaining the MADS, I (intervening) and K‐box domains were generated for all seven apple genes using gene‐specific primers (Table S1), and the sequences obtained were cloned into *pDONR201*. All vectors were confirmed by sequencing.

### Complementation assay in Arabidopsis

For details regarding plasmid construction, see Methods S1. All constructs were introduced into *Agrobacterium* strain GV3101 (Hood *et al*., 1993), and Arabidopsis *svp‐41* mutant plants (Hartmann *et al*., 2000) were transformed using the floral dip method (Clough & Bent, 1998). T1 independent lines were BASTA selected, lines showing a Mendelian segregation (3 : 1) were retained, and two to three homozygous single‐copy T3 lines from independent T1 lines were selected for further use. Seeds were stratified on soil in the dark at 4°C for 7 d. Plants were grown under controlled conditions at 22°C with a long‐day (LD) photoperiod (16 h : 8 h, light : dark). Arabidopsis Columbia‐0 (Col‐0) was used as the wild‐type (WT). Flowering time was scored by counting total leaf number (cauline and rosette leaves) of at least 10 plants per genotype. The number of days from germination to bolting (elongation of the first internode by 0.5 cm) and to the opening of the first flower were recorded. Total RNA was isolated from 7‐d‐old manually dissected leaves and shoot apices using the RNeasy Mini Kit (Qiagen). Real‐time polymerase chain reaction was performed as previously described. *PP2A* was used as a reference gene.

### Yeast two‐hybrid assays

Full‐length and truncated gene versions were recombined into *pDEST22* and *pDEST32* vectors (Invitrogen). Protein–protein interactions were tested by transforming the yeast PJ69‐4A strain using the Frozen‐EZ Yeast Transformation II (Zymo Research, Irvine, CA, USA) protocol. Yeast selection was initially conducted in SD plates lacking Leu and Trp (SD–LW). Three to five colonies were randomly mixed and grown on SD–LW plates or SD plates lacking Leu, Trp and His (SD–LWH) with or without the addition of 3‐amino‐1,2,4‐triazole (3‐AT). The yeast were grown at 30°C for 6 d.

### Tobacco co‐immunoprecipitation

Genes were recombined into N‐terminal 5xMyC or green fluorescent protein (GFP) using the pAM backbone (Zhou *et al*., 2016), and transformed into *Agrobacterium*. Protein combinations were co‐infiltrated into *Nicotiana benthamiana* leaves as described previously (Fernández *et al*., 2016). For details concerning protein extraction and immunoprecipitation, see Methods S1. Western blotting was performed to detect immunoprecipitated proteins using anti‐GFP antibody (ab290; Abcam, Cambridge, MA, USA) and co‐immunoprecipitated with anti‐MyC antibody (9E1; Chromotek, Planegg‐Martinsried, Germany) as described previously (Fernández *et al*., 2016). Chemiluminescence detection of proteins was performed using the ChemiDoc MP Imager (Bio‐Rad).

### Confocal microscopy

Arabidopsis leaves (7‐d‐old) and shoot meristems (10‐d‐old) were prepared as described previously (Kurihara *et al*., 2015; Sang *et al*., 2020). Samples were imaged using a confocal laser scanning microscope (SP8; Leica, Wetzlar, Germany) with settings optimized to visualize Venus (laser wavelength, optically pumped semiconductor laser (OPSL) 514 nm; detection wavelength, 521–531 nm) and Renaissance 2200 (laser wavelength, diode 405 nm; detection wavelength, 424–478 nm). To visualize GFP (laser wavelength, OPSL 488 nm; detection wavelength 492–517 nm) in tobacco leaves, the abaxial side of the leaves were imaged 3 d post‐inoculation.

### Electrophoretic mobility shift assay (EMSA) and seq‐DAP‐seq experiments

For details related to plasmid construction, and *in vitro* co‐production and sequential purification of proteins, please refer to Methods S1. The EMSA was performed as described previously (Hugouvieux *et al*., 2018), using a 103 bp fragment of the Arabidopsis *SEP3* promoter containing two CArG boxes as a DNA probe (10 nM). The AG–SEP3 complex was used as a positive control. Sequential DNA‐affinity purification sequencing was performed essentially in the same manner described in a previous study by Lai *et al*. (2020). Thirteen seq‐DAP‐seq libraries were generated using apple genomic DNA extracted from Gala endodormant buds. Libraries with different barcodes (NEBNext Multiplex Oligos for Illumina, Ipswich, MA, USA) were pooled with equal molarity, and sequenced on an Illumina HiSeq (GENEWIZ, South Plainfield, NJ, USA) with specification of paired‐end sequencing of 150 bp. Each library obtained between 40 and 60 million reads (730 million reads total).

### Calli transformation and RNA‐seq

Genes fused to the GR were recombined into the *pCamway35S* binary vector (Leclercq *et al*., 2015), followed by *Agrobacterium* transformation. Transformed calli were obtained from apple leaf explants and dexamethasone (DEX) treatment was performed as described in a previous study by Estevan *et al*. (2020) and Methods S1. RNA was isolated using the Spectrum Plant Total RNA kit (Sigma‐Aldrich). Samples, in triplicate, were used to generate 24 Illumina Truseq Stranded mRNA libraries that were sequenced using the GeT Platform (https://get.genotoul.fr/en/) with an Illumina HiSeq 3000 and a specification of paired‐end sequencing of 150 bp. Each library obtained between 20 and 40 million reads (670 million reads total).

### Bioinformatic analyses

Detailed methods for seq‐DAP‐seq, RNA‐seq and gene ontology (GO) enrichment analyses are provided in Methods S1.

### Statistical analysis

Statistical analysis using Kruskal–Wallis one‐way ANOVA, ANOVA and *t*‐tests were conducted using Prism 5.0a (GraphPad, www.graphpad.com). Fisher’s exact test and the hypergeometric test were conducted using R.

## Results

### *MdSVPa* and *MdSVPb* but not *MdDAM*‐like genes complement the early‐flowering phenotype of Arabidopsis *svp‐41*

**Fig. 1:**
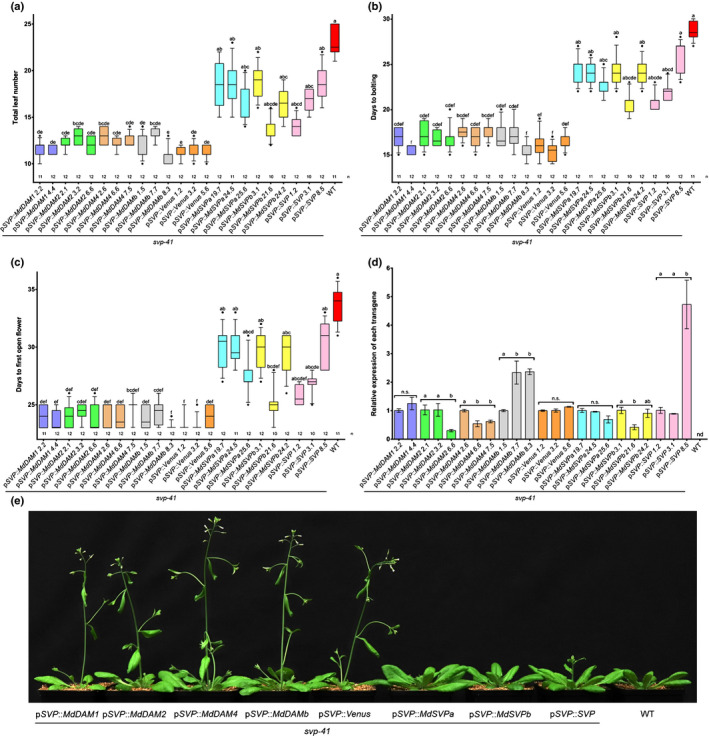
Complementation assay of the early‐flowering phenotype of the Arabidopsis *svp*‐*41* mutant under long‐day conditions. (a) Total leaf number (including cauline and rosette leaves) was scored before flowering. (b) Number of days from germination to bolting (elongation of the first internode by 0.5 cm). (c) Number of days from germination to the opening of the first flower. In (a) to (c), the box extends from the 25^th^ to 75^th^ percentiles, the line in the middle is plotted at the median, and the whiskers are drawn down to the 10^th^ and up to the 90^th^ percentile. The outliers below and above the whiskers are drawn as individual points. Kruskal–Wallis one‐way ANOVA followed by Dunn’s test was used to test statistical significance. Lowercase letters shared by genotypes indicate no significant differences (for *P* ≤ 0.05). (d) Relative expression of the different transgenes after 7 LDs. Gene expression from three independent biological replicates ± SEM is shown relative to the reference gene *PP2A*, with the first genotype of each transgene set to 1. Statistical analysis was performed using one‐way ANOVA followed by Tukey’s multiple comparison test or a *t*‐test for *MdDAM1* comparisons. Lowercase letters shared by genotypes indicate no significant differences (for *P* ≤ 0.05). nd, not detected; ns, not significant. (e) Plants representing intermediate phenotypes of each genotype grown under long‐day conditions for 25 d. WT, wild‐type.

Previous phylogenetic analysis has shown that Rosaceous species carry two groups of *SVP*‐related genes: a cluster solely composed of Rosaceous *DAM* genes and another well‐defined cluster formed by Arabidopsis *SVP* and *SVP*‐like genes from Rosaceous species (Falavigna *et al*., 2019). This classification will be used to describe the genes under study here. Apple contains five *DAM*‐like and two *SVP*‐like genes. The coding sequences (CDSs) of four out of five *DAM*‐like genes and both *SVP*‐like genes were amplified and cloned. To evaluate the conservation of their protein function with that of *SVP*, these apple genes were heterologously expressed in an Arabidopsis mutant lacking *SVP* function (*svp‐41*), which exhibits an early‐flowering phenotype (Hartmann *et al*., 2000). For this purpose, 3 kb of the *SVP* promoter was used to drive transgene expression. The Arabidopsis *SVP* gene was used as a positive control (Gregis *et al*., 2009), and the Venus fluorescent protein was used to monitor the temporal and spatial expression pattern conferred by this promoter. T1 transformants were scored for flowering time based on the number of rosette leaves at bolting (Fig. S1a). Homozygous single‐copy T3 lines were obtained and scored for flowering‐time traits under long‐day (LD) conditions. Plants carrying Venus alone did not complement *svp*‐*41* (Fig. S1b). Confocal microscopy of these plants showed that the *SVP* promoter used in this study correctly guided the expression of the transgenes throughout expanded leaves and the SAM (Fig. S2) (Hartmann *et al*., 2000; Lee *et al*., 2007; Jang *et al*., 2009). Plants expressing *SVP*, *MdSVPa* and *MdSVPb* were reproducibly late flowering under LD conditions in comparison to *svp*‐*41* (Fig. 1a–c,e). Lines showing an intermediate phenotype contained lower transgene mRNA levels than their counterparts (Fig. 1d). Lines carrying *MdDAM*‐like genes showed transgene mRNA levels similar to those observed in plants expressing *SVP*, *MdSVPa* and *MdSVPb*. However, they could not complement any of the *svp*‐*41* floral phenotypes. No statistical significance was observed for any of the flowering traits analyzed among plants expressing *SVP*, *MdSVPa* or *MdSVPb*, which suggests that apple SVP‐like proteins share conserved functions with Arabidopsis SVP, whereas MdDAM proteins do not. These data suggest an evolutionary diversification between *DAM*‐ and *SVP*‐like genes within the Rosaceae.

### *MdSVPa* interacts with several apple *MADS* transcription factors related to dormancy‐specific phases

**Fig. 2:**
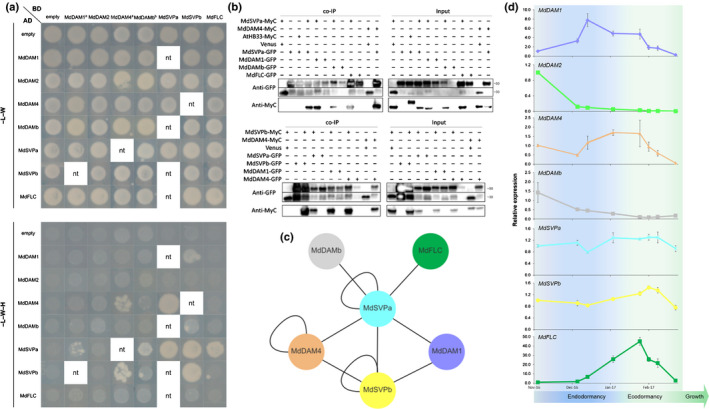
Protein interaction and gene expression analysis of genes encoding apple DAM‐, SVP‐ and FLC‐like proteins. (a) Yeast two‐hybrid assay of truncated apple MADS proteins. Interactions were reciprocally tested unless a positive interaction was identified beforehand. In this case, the other direction was not tested (blank spaces with nt – not tested). The negative controls are representative pictures of several independent controls. ^a^MdDAM1 and MdDAM4 fused to the DNA‐binding domain were evaluated in SD–LWH media (i.e. without Leu, Trp and His) supplemented with 0.5 mM 3‐AT. ^b^The interaction between MdSVPa and MdDAMb was observed using full‐length protein versions. See Supporting Information Fig. S3 for further information. (b) *In planta* co‐immunoprecipitation of protein interactions detected by yeast two‐hybrid assay. MdDAM4, MdSVPa and MdSVPb were translationally fused to MyC, whereas MdDAM1, MdDAM4, MdDAMb, MdSVPa, MdSVPb and MdFLC were translationally fused to green fluorescent protein (GFP). Protein–protein interactions were tested in pairs by agroinfiltration of tobacco leaves. The input was composed of total proteins recovered before immunoprecipitation. GFP‐fused proteins were immunoprecipitated using anti‐GFP nanobody (VHH) beads and immunoblotted using anti‐MyC or anti‐GFP antibody. Controls were used to ensure that the MADS transcription factors (TFs) did not recognize and bind to GFP‐ or MyC‐tag (Venus fused to a nuclear localization signal (NLS) and AtHB33‐MyC, respectively). The anti‐GFP blot was split in two due to differences in protein size between NLS‐Venus (*c*. 30 kDa) and the GFP fusions (*c*. 50 kDa). (c) Summary of the protein–protein interactions identified in this work. (d) Relative expression of apple *DAM*‐, *SVP*‐ and *FLC*‐like genes during the dormancy cycle. Gene expression from three independent biological replicates ± SEM is shown relative to the reference genes *MdMDH* and *MdWD40*, with the first sampling point of each gene set to 1. Transition from endo‐ to ecodormancy occurred in mid‐to‐late January (Fig. S6).

Dimerization is a key feature of MADS TFs (de Folter *et al*., 2005; Smaczniak *et al*., 2017), and we tested whether apple DAM‐ and SVP‐like proteins form complexes. The apple FLC‐like (MD09G1009100, (Porto *et al*., 2015)) protein was included due to the well‐characterized SVP–FLC module in Arabidopsis (Mateos *et al*., 2015). Full‐length coding sequences and truncated versions lacking their C terminus to abolish autoactivation of yeast reporter genes (Immink *et al*., 2002; Leseberg *et al*., 2008) were used to screen protein–protein interactions using yeast two‐hybrid assays (Figs 2a, S3). MdDAM1 and MdDAM4 interacted with MdSVPb. MdDAM4, MdSVPa and MdSVPb proteins formed homodimers. All tested proteins interacted with MdSVPa, except MdDAM2, which did not interact with any of the tested proteins. Co‐immunoprecipitation assays were carried out in agroinfiltrated *N*. *benthamiana* leaves to test whether these interactions also occur *in planta* (Fig. 2b). All protein–protein interactions observed in the yeast two‐hybrid assay were validated, which demonstrates a physical interaction network among apple DAM‐, SVP‐ and FLC‐like proteins (Fig. 2c). Notably, MdSVPa appeared as a central hub among these interacting proteins. Additionally, all apple proteins were localized to the nucleus (Fig. S4), providing further evidence that the protein–protein interactions identified here may have a transcriptional regulatory role.

Next, we investigated the function of apple MADS TFs during dormancy. The transcript levels of their encoding genes were quantified from endodormancy establishment to ecodormancy release (Fig. 2d). *MdDAM1* and *MdDAM4* expression peaked in the middle of endodormancy, with decreasing levels after ecodormancy. *MdDAM2* and *MdDAMb* presented low expression levels after endodormancy establishment. *MdFLC* transcript levels were highest during the transition from endo‐ to ecodormancy. Finally, *MdSVPa* and *MdSVPb* were expressed almost at constant levels during dormancy. These results suggest that MdSVPa is part of transcriptional complexes in combination with MADS TFs that are predominant at dormancy‐specific phases.

### MdSVPa complexes bind to and regulate the expression of hundreds of genes

**Fig. 3:**
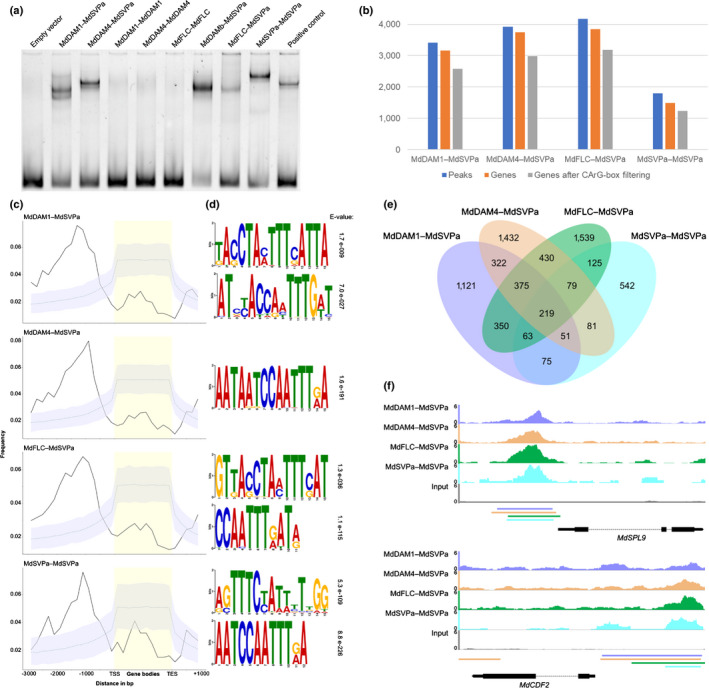
Genome‐wide target sites of transcriptional complexes containing MdSVPa. (a) Electrophoretic mobility shift assay (EMSA) evaluating the ability of homo‐ and heterodimers to recognize a DNA probe consisting of a 103‐bp fragment of the Arabidopsis *SEP3* promoter containing two CArG boxes (Hugouvieux *et al*., 2018). The Arabidopsis AG–SEP3 complex was used as a positive control, and the band obtained represents the size of a heterotetramer. (b) Number of seq‐DAP‐seq peaks, genes associated with peaks and genes obtained after filtering for the presence of at least one CArG‐box. (c) Peak distribution in a region from 3 kb upstream of the transcription start site (TSS) to 1 kb downstream of the transcription end site (TES) of the closest gene. Solid lines represent the positional distribution of observed peaks for each complex. Dashed lines correspond to the observed positional distribution obtained from 1000 randomly generated peak sets. As the distributions were determined using bins, the 95% confidence interval (gray shaded color) for each bin is depicted. (d) Logos of the enriched sequence motifs identified by Multiple Em for Motif Elicitation (MEME) motif analysis. The *E*‐value indicates the statistical significance for the corresponding motif. (e) Venn diagram illustrating common genes between the four seq‐DAP‐seq datasets after filtering for the presence of CArG boxes. (f) DNA‐binding profiles of the four complexes and the control (input) at the promoter regions of *MdSPL9* and *MdCDF2*. Horizontal bars below the plots represent the positions of the peak regions. The color code between the plots and the bars is preserved. The Integrated Genome Browser (IGB) was used for visualization.

The biological relevance of the identified MADS TF complexes was tested in Arabidopsis *MdSVPa*/*MdDAM* double transgenics. No additive flowering phenotypes were observed in F1 lines heterologously expressing both transgenes vs single *MdSVPa* transgenics (Fig. S5), indicating that MdDAM and MdSVP TFs have different functions, at least in the context of flowering‐time control of Arabidopsis. We then made use of seq‐DAP‐seq (Bartlett *et al*., 2017; Lai *et al*., 2020) to elucidate the function of these MADS complexes by studying their genome‐wide binding sites in apple. MdDAM2 and MdDAMb were not included in this experiment because the genes encoding their proteins showed low expression levels after endodormancy establishment (Fig. 2d). Protein complexes were co‐produced by coupled *in vitro* transcription/translation and visualized using Western blots after co‐immunoprecipitation (Fig. S7a). Strikingly, only complexes containing MdSVPa exhibited any DNA binding in EMSA experiments and these complexes were used in subsequent seq‐DAP‐seq studies (Fig. 3a). Purified protein complexes were incubated with an apple genomic DNA library obtained during dormancy. DNA fragments were recovered, sequenced using massive parallel sequencing, and aligned to the apple genome.

Merged peaks for each transcriptional complex were called and assigned to neighboring genes (Fig. 3b, Dataset S1). Genomic enrichment upstream of the transcription start site (TSS) was observed for all complexes, consistent with their function as TFs (Fig. 3c). *De novo* motif discovery detected an enrichment of two putative CArG boxes with similar nucleotide composition but different numbers of consecutive nucleotides in the central A/T‐rich region (Fig. 3d). Sequences similar to the canonical CArG‐box (CC(A/T)_6_GG) were observed for the MdDAM1–MdSVPa, MdFLC–MdSVPa and MdSVPa–MdSVPa complexes, whereas a  CArG‐box with 5 bp length in its A/T stretch region (CC(A/T)_5_GG) was recurrently detected for all complexes. CArG boxes with variable A/T‐length content has been reported previously for MADS TFs (Kaufmann *et al*., 2009; Smaczniak *et al*., 2017), including SVP and FLC (Mateos *et al*., 2015). Complementarily, a manual search for CArG‐box motifs was performed, and 80% of the peaks mapped close to gene models contained at least one CArG‐box, which is a much higher frequency than expected for a random distribution (Figs 3b, S7b–c; Dataset S2).

After the CArG‐box filtering, 55% of the targets were common to at least two complexes (Fig. 3e). Gene ontology term analysis showed an enrichment for the targets of all four complexes in categories related to response to endogenous and abiotic stimuli, cellular processes, carbohydrate metabolic processes, multicellular organism development and anatomical structure development (Fig. S7d). A total of 219 targets were bound by all four complexes, including important flowering‐time regulators such as *SQUAMOSA PROMOTER‐BINDING PROTEIN‐LIKE 9* (*MdSPL9*, MD14G1060200) and *CYCLING DOF FACTOR 2* (*MdCDF2*, MD16G1071400; Fig. 3f). Moreover, *MdDAM1* was bound by MdDAM4–MdSVPa, MdFLC–MdSVPa and MdSVPa–MdSVPa, whereas *MdFLC* was bound by MdDAM1–MdSVPa and MdSVPa–MdSVPa (Fig. S7e).

**Fig. 4:**
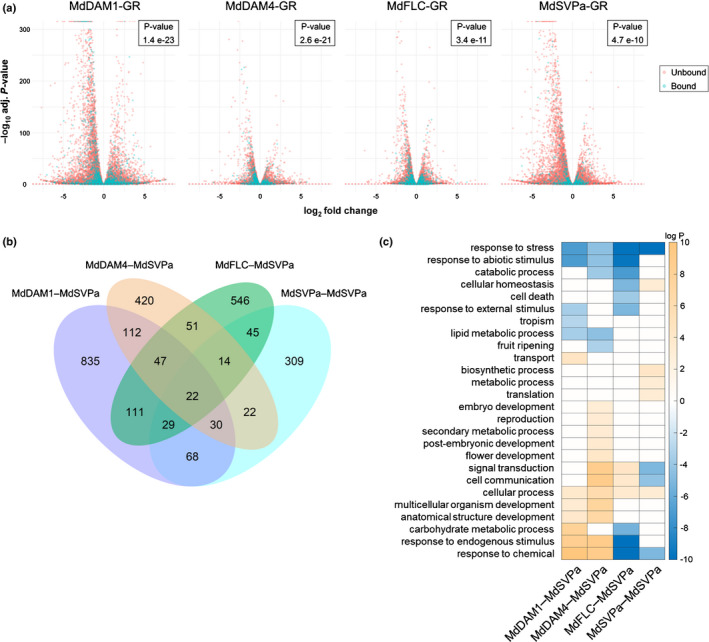
Characterization of the target genes from four transcriptional complexes containing MdSVPa. (a) Volcano plots representing differentially expressed genes (DEGs) identified using the GR DEX‐inducible system in transgenic apple calli transformed with four apple transcription factors (TFs). Bound genes represent genes also identified using the seq‐DAP‐seq technique. The inset image shows the *P*‐value (one‐sided Fisher’s exact test) obtained when analyzing the statistical significance of the overlap between target genes identified using seq‐DAP‐seq and the DEX‐inducible assay. (b) Venn diagram illustrating common genes between the high‐confidence targets of each complex. (c) Gene ontology (GO) term enrichment analysis of the high‐confidence targets of each transcriptional complex containing MdSVPa. Enrichment tests were performed separately for up‐ and downregulated gene sets and only the best *P*‐value (smallest) was kept. For data visualization, the best *P*‐value was transformed using –log or log when it belonged to the up (orange gradient) or downregulated (blue gradient) gene set, respectively. Note that all *P*‐values > 0.05 were replaced by zero (white boxes).

To evaluate the transcriptional changes of direct targets of these four MADS TFs in apple, calli were transformed with constructs carrying MdDAM1, MdDAM4, MdFLC and MdSVPa fused to GR. Positive transformed calli for each construct were pretreated with cycloheximide, followed by DEX or solvent (mock) treatments (Aoyama & Chua, 1997). RNA‐seq analysis compared DEX and mock treatments after calli were incubated for 8 h at room temperature (Fig. S8a). After DEX induction, differentially expressed genes (DEGs, for adj. *P* ≤ 0.05) were identified (Fig. 4a; Dataset S3) and 79–91% of the DEGs were common to at least two MADS TFs (Fig. S8b). Notably, *MdSVPa* was expressed at high levels in all transformed calli (NCBI SRA PRJNA698061). *MdDAM1* was repressed by MdDAM1‐GR and MdSVPa‐GR, whereas *MdSVPa* was slightly induced by MdDAM1‐GR. *MdDAM4* and *MdFLC* showed autoregulation, being repressed and induced by their encoded TFs, respectively. A GO analysis was performed, and enriched terms were classified as up‐ or downregulated based on the expression of the genes that composed each category (Fig. S8c). Several GO categories showed differential regulation between the four MADS TFs, such as signal transduction, cell communication, carbohydrate metabolic processes and metabolic processes.

A statistically significant overlap (one‐sided Fisher’s exact test, inset in Fig. 4a) was obtained between the seq‐DAP‐seq data and the DEGs from the GR DEX‐inducible assay for all four comparisons. The genes found in the intersection between both assays were considered to be high‐confidence targets of each complex (Fig. 4b; Dataset S4). Gene ontology term analysis, coupled with analysis of the direction of gene regulation (i.e. up‐ or downregulation), was conducted, considering the high‐confidence targets of each complex. This analysis revealed that, even though some GO categories showed similar enrichment among complexes, each MADS transcriptional complex differentially regulates plant processes (Fig. 4c).

### Clusters with similar expression profiles but regulated by different *MdSVPa*‐containing complexes have distinct biological functions during dormancy

**Fig. 5:**
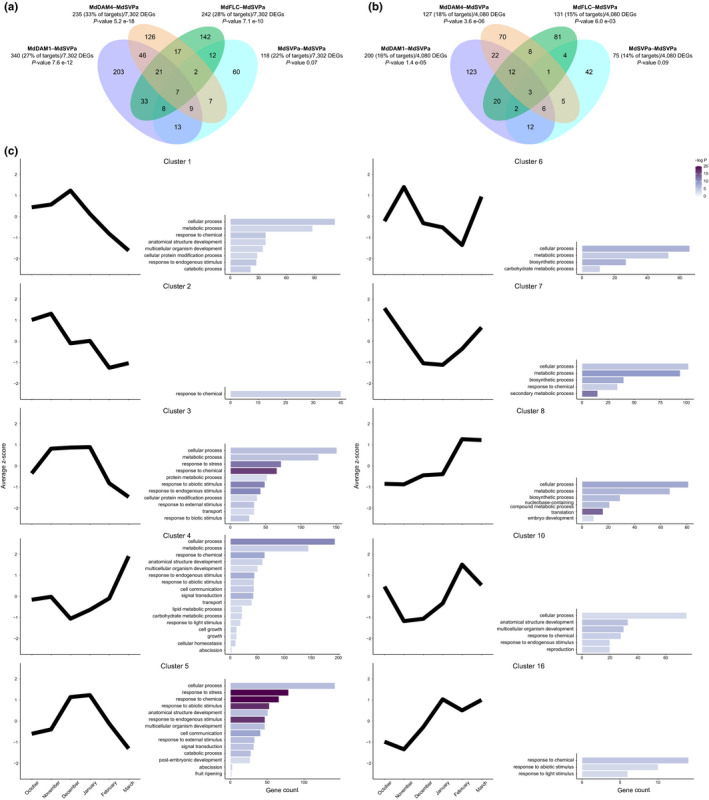
Comparisons between dormancy‐related differentially expressed genes (DEGs) and the target genes of four transcriptional complexes containing MdSVPa. (a) Venn diagram showing the overlap between dormancy‐related DEGs identified in field‐grown samples (dataset A) and the high‐confidence target genes of each transcriptional complex. (b) Venn diagram displaying the overlap between dormancy‐related DEGs identified under controlled conditions (dataset B) and the high‐confidence target genes of each transcriptional complex. The lists of target genes from each transcriptional complex were evaluated for the enrichment of dormancy‐related DEGs, and the *P*‐value obtained (using the hypergeometric test) is shown. (c) Gene expression and gene ontology (GO) enrichment analysis of the high‐confidence target genes of MdDAM1, MdDAM4 and MdFLC in association with MdSVPa during dormancy (Supporting Information Dataset S5). Average *z*‐score values for clusters were obtained from an annual time course expression analysis of apple buds harvested from field‐grown ‘Golden delicious’ trees (dataset A). Only clusters showing at least one significant GO term (*P* < 0.05) are represented. The colormap represents the −log_10_ *P*‐value.

We tested whether the high‐confidence targets of each complex were enriched for genes with differential expression during dormancy. We re‐analyzed two RNA‐seq experiments performed with apple buds harvested in the field (‘dataset A’ hereafter) (Moser *et al*., 2020) and controlled conditions (‘dataset B’) (Takeuchi *et al*., 2018). In these experiments, *MdDAM1*, *MdDAM4*, *MdFLC* and *MdSVPa* showed similar expression profiles to those observed in our expression assays (Figs 2d, S9a,b). A total of 7302 and 4080 DEGs (for adj. *P* ≤ 0.05 and log2fold change < or > 1.75) were identified in datasets A and B, respectively. Notably, the high‐confidence targets of MdDAM1–MdSVPa, MdDAM4–MdSVPa, and MdFLC–MdSVPa were enriched with DEGs from datasets A and B (hypergeometric test, for *P* ≤ 0.05; Fig. 5a,b). Conversely, target genes from the MdSVPa homodimer were not enriched with DEGs during dormancy in any dataset, which suggests that the role of MdSVPa as regulator of dormancy requires its association with other MADS TFs.

To further study the role of heteromeric complexes during dormancy, we generated a list containing all target genes of MdDAM1, MdDAM4 and MdFLC in transcriptional complexes with MdSVPa (2356 unique genes) and analyzed their expression dynamics during dormancy (dataset A). Based on their expression patterns, 17 clusters were identified (Dataset S5). Only cluster 4 contained GO terms associated with growth, cell growth and cellular homeostasis, whereas terms associated with response to endogenous and abiotic stimuli, abscission, signal transduction and cell communication were also enriched in cluster 5, and clusters 1 and 3 to some extent. Interestingly, while genes from cluster 4 were repressed during endodormancy, these three clusters showed the opposite expression pattern during dormancy (i.e. a peak of expression during endodormancy; Fig. 5c). Taken together, these data suggest that the association of MdSVPa with other MADS TFs has a regulatory role in several plant processes occurring during dormancy. However, the MdDAM1–MdSVPa, MdDAM4–MdSVPa, and MdFLC–MdSVPa complexes showed a high number of unique dormancy‐related DEGs (Fig. 5a,b), suggesting that each complex is regulating different processes.

**Fig. 6:**
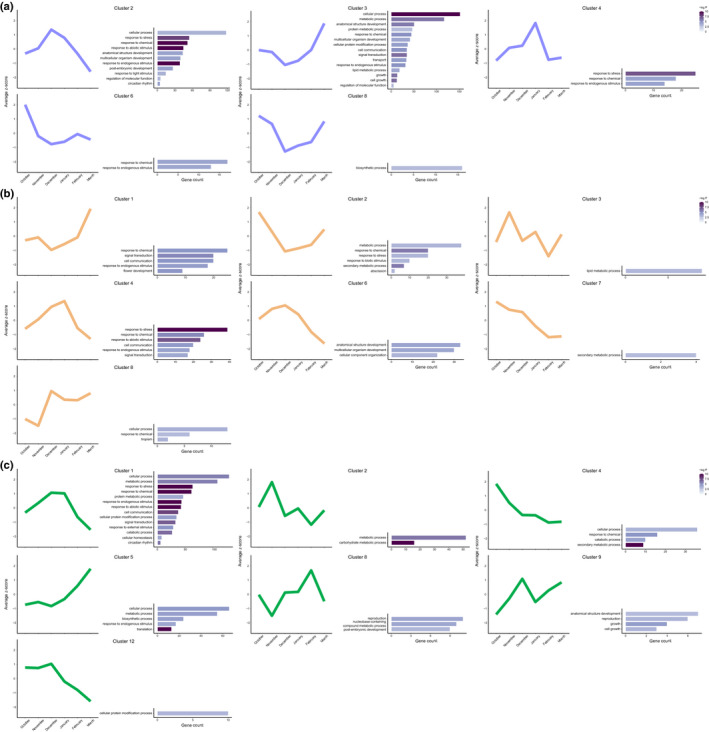
Average expression patterns and gene ontology (GO) enrichment analysis of the different clusters obtained during apple bud dormancy. (a) Analysis of the high‐confidence target genes of MdDAM1–MdSVPa during dormancy (Supporting Information Dataset S6). (b) Analysis of the high‐confidence target genes of MdDAM4–MdSVPa during dormancy (Dataset S7). (c) Analysis of the high‐confidence target genes of MdFLC–MdSVPa during dormancy (Dataset S8). Average *z*‐score values for clusters were obtained from an annual time course expression analysis of apple buds harvested from field‐grown ‘Golden delicious’ trees (dataset A). Only clusters showing at least one significant GO term (*P*‐value < 0.05) are represented. The colormap represents the −log_10_ *P*‐value.

Subsequently, we analyzed the individual contributions of the MdDAM1–MdSVPa, MdDAM4–MdSVPa, and MdFLC–MdSVPa complexes to dormancy control. Based on the expression patterns of MdDAM1–MdSVPa targets during dormancy (dataset A), 11 clusters were identified (Dataset S6) and five of them were enriched with GO terms (Fig. 6a). Clusters 2 and 3 shared GO terms associated with cellular processes, regulation of molecular function, anatomical structure development, and response to endogenous stimulus. However, these clusters presented antagonistic patterns of expression, as cluster 2 showed high expression levels during endodormancy, while cluster 3 was repressed at the same time points. Additionally, cluster 2 was enriched in terms related to circadian rhythm, post‐embryonic development, and response to abiotic and light stimuli. Conversely, cluster 3 showed terms associated with growth, cell communication, and signal transduction. The target genes of MdDAM4–MdSVPa were grouped in nine expression clusters (Fig. 6b; Dataset S7). Genes belonging to cluster 1 were induced near budbreak, with low levels during endodormancy. This cluster was enriched with terms associated with response to endogenous stimulus, signal transduction, cell communication, and flower development. Cluster 4 was enriched in the same GO terms – except for the flower development‐related terms, which were absent, and the addition of terms related to response to stress and abiotic stimulus – but its genes showed a peak in transcription during endodormancy. Finally, the high‐confidence targets of MdFLC–MdSVPa were classified in 12 clusters during dormancy (Dataset S8), and GO analysis found that seven of them were enriched (Fig. 6c). Genes composing cluster 1 were upregulated during endodormancy, and categories associated with circadian rhythm, signal transduction, cell communication, and response to external, endogenous and abiotic stimuli were found to be enriched. A more detailed GO enrichment analysis focusing on biological processes revealed further differences between MADS complex targets and expression clusters (Dataset S9). These results suggest that each transcriptional complex is able to co‐regulate genes in a similar manner, but these clusters are composed of different sets of genes and thus may have different biological functions during dormancy.

### MADS transcription factor complexes act in concert to fine‐tune the dormancy cycle in apple

Next, we focused on identifying genes which play roles during dormancy and act downstream of the MADS transcriptional complexes. A significant overlap was identified between the DEGs from datasets A and B (hypergeometric test, for *P* ≤ 0.05; Fig. S9c), and a list of DEGs found in the intersection between both analyses was generated (dormancy‐related DEGs, Dataset S10). Subsequently, the overlap between the dormancy‐related DEGs and the high‐confidence targets of the MdDAM1–MdSVPa, MdDAM4–MdSVPa, and MdFLC–MdSVPa complexes (Dataset S11) was obtained, and some of these genes were further characterized based on their putative role in dormancy regulation. The DNA‐binding profiles of the MADS complexes to the locus region of 11 dormancy‐related genes, and the way in which their gene expression is regulated by the complexes, are depicted in Fig. S10. *MdBRC1* (*BRANCHED 1*; MD06G1211100) and *MdMP* (*MONOPTEROS*; MD15G1014400) were bound and induced by the MdDAM1–MdSVPa and MdDAM4–MdSVPa complexes (Fig. S10a). Both genes are known to be part of GRNs regulating bud and meristem development (Luo *et al*., 2018; Singh *et al*., 2018; Wang *et al*., 2019; Maurya *et al*., 2020; Cucinotta *et al*., 2021). *MdBRC1* expression peaked during endo‐ to ecodormancy transition, maintaining high levels until budbreak (Fig. 5c, cluster 16), whereas *MdMP* only showed transcriptional induction near budbreak (Fig. 5c, cluster 8). In spite of both genes being common targets of the same transcriptional complexes, they showed a differential temporal regulation of expression during dormancy, which highlights the complex gene regulation that occurs during this phase.

Genes related to hormone biosynthesis and signaling were also present in this list. *MdCKX5* (cytokinin oxidase/dehydrogenase 5; MD15G1021500), which in Arabidopsis regulates the activity of the reproductive meristems by catalyzing the oxidation of cytokinins (CKs) (Bartrina *et al*., 2011), was upregulated during endodormancy (Fig. 5c, cluster 3), and was bound and transcriptionally activated by the MdDAM1–MdSVPa and MdFLC–MdSVPa complexes (Fig. S10a). Recently, it was proposed that CK stimuli repress *MdDAM1* expression (Cattani *et al*., 2020), suggesting that a negative feedback loop between MADS TFs and CKs may exist. *MdGA2ox1* (*GA2‐oxidase 1*; MD05G1207000) encodes an enzyme responsible for the deactivation of bioactive gibberellin (GA), preventing the accumulation of this growth‐promoter hormone during dormancy (Yamaguchi, 2008; Beauvieux *et al*., 2018; Fadón *et al*., 2020). This gene was bound and repressed by the MdFLC–MdSVPa complex (Fig. S10a). *MdNCED4* (*9‐cis‐epoxycarotenoid dioxygenase 4*; MD16G1090700) and *MdPYL4* (*pyrabactin resistance‐like 4*; MD07G1227100) are involved in abscisic acid (ABA) biosynthesis and signaling, respectively, and showed opposite expression trends during dormancy. *MdNCED4* was repressed after dormancy establishment (Fig. 5c, cluster 4), while *MdPYL4* was induced during endodormancy (Fig. 5c, cluster 3). Likewise, *MdNCED4* was repressed by MdDAM1–MdSVPa and *MdPYL4* was induced by MdFLC–MdSVPa (Fig. S10a). We have also observed the presence of genes related to cell wall modification processes. The involvement of xyloglucan metabolism during the dormancy cycle has been reported previously (Čechová *et al*., 2012; Viti *et al*., 2013; Saito *et al*., 2015; Zhao *et al*., 2020). *MdXTH9* (*xyloglucan endotransglucosylase/hydrolase*; MD09G1102600), *MdXTH15* (MD09G1152700) and *MdXTH23* (MD13G1237300), three genes encoding enzymes related to loosening and rearrangement of the cell wall, were induced by the MdDAM1–MdSVPa complex (Fig. S10a). Conversely, *MdXTH15* was repressed by the other MADS complexes.

Finally, genes related to flowering‐time regulation through the photoperiodic pathway were also selected. *MdCDF2* and *MdLHY* (*LATE ELONGATED HYPOCOTYL*, MD01G1090900) were upregulated during endodormancy, showing lower expression levels near budbreak (Fig. 5c, cluster 3). *MdCDF2* was bound and repressed by MdDAM4–MdSVPa, whereas *MdLHY* was induced by MdDAM1–MdSVPa and repressed by MdDAM4–MdSVPa (Figs 3f, S10a). In Arabidopsis, LHY and CDFs act in overlapping flowering pathways (Mizoguchi *et al*., 2002; Renau‐Morata *et al*., 2020), leading to the activation and accumulation of CONSTANS (CO), which further activates the expression of the floral integrator gene *FLOWERING LOCUS T* (*FT*) and, consequently, *SUPPRESSOR OF OVEREXPRESSION OF CONSTANS 1* (*SOC1*) (Andrés & Coupland, 2012; Wang *et al*., 2021). Interestingly, *MdSOC1a* (MD02G1197400), an ortholog of the Arabidopsis *SOC1* gene, was bound and repressed by the MdSVPa homomeric complex (Fig. S10b). In Arabidopsis, SVP represses *SOC1* expression as a part of a GRN that regulates floral transition in the SAM (Li *et al*., 2008). The SVP‐SOC1 regulatory module seems to be conserved in apple, although its implication in flowering and/or dormancy control remains unknown.

## Discussion

The finely tuned control of dormancy is fundamental for temperate fruit trees, to ensure their survival during winter while being ready to maximize their reproductive cycle after budbreak. Recent genetic studies have revealed that DAM‐ and SVP‐like MADS TFs are master regulators of the dormancy cycle; however their target genes and GRNs were not known. Here, we addressed how the different MADS complexes integrate GRNs to shape dormancy dynamics by employing seq‐DAP‐seq for the first time in nonmodel species. Moreover, by coupling this genome‐wide binding data to GR DEX‐inducible assays, we managed to overcome many of the difficulties associated with performing functional genetics in fruit tree species, thus providing an experimental pipeline that is easily adaptable to any target TF complex and species.

### The neofunctionalization of the DAM genes could have caused their dormancy specialization in fruit trees

DAM TFs are phylogenetically related to Arabidopsis SVP and, therefore, are often referred to as SVP‐like. However, Rosaceous DAM TFs belong to a specific clade that is separate from the cluster containing the Arabidopsis SVP, which also includes MdSVPa and MdSVPb (Liu *et al*., 2018, 2020; Falavigna *et al*., 2019). The divergence between DAM and SVP is partially caused by structural changes in the DNA‐binding MADS domain (Norman *et al*., 1988; Ma *et al*., 1991), suggesting functional diversification between these two groups. The heterologous expression of *MdDAM* and *MdSVP* genes under the control of the Arabidopsis *SVP* promoter showed that only *MdSVPa* and *MdSVPb* restore the WT flowering phenotype of an *svp* null Arabidopsis mutant (Fig. 1). We ruled out the possibility that the lack of flowering activity of MdDAM TFs was due to their inability to form functional complexes in the absence of *SVP* (Fig. S5). These results indicate that apple and Arabidopsis *SVP* genes retained an ancestral flowering repression function that the apple *DAM* genes have lost during evolution. Moreover, whereas the central role of SVP TFs as a part of transcriptional complexes that regulate developmental processes seems to be conserved across taxa, DAM TFs might have evolved to regulate dormancy‐specific functions in Rosaceae. This dormancy‐related function is believed to be similar to the role that FLC plays in the control of flowering mediated by vernalization in Arabidopsis (Ríos *et al*., 2014; Mateos *et al*., 2017). It would not be surprising to discover that the dormancy cycle is controlled by FLC‐like TFs in trees. However, this seems not to be the case for the *FLC*‐like gene studied in this work, at least, as its expression pattern suggests a role related to ecodormancy and independent of cold requirement (Fig. 2d) (Porto *et al*., 2015; Takeuchi *et al*., 2018). Moreover, during Rosaceae evolution, *SVP*‐like genes were expanded and several *FLC*‐like genes were lost (Liu *et al*., 2020), favoring the neofunctionalization of some *SVP*‐like genes to take dormancy‐related roles, thus explaining the evolutionary origin of the MdDAM TFs.

A subsequent subfunctionalization event within the *DAM* genes could have provided more flexibility to the GRNs controlling dormancy in fruit trees. This could be due to the differential expression patterns shown by the *DAM* genes, as previously proposed (Jiménez *et al*., 2009; Li *et al*., 2009), but also by the combination of DAM TFs in transcriptional complexes with distinct DNA‐binding preferences, as shown by our seq‐DAP‐seq data (Figs 3, S7, S10). Interestingly, Rosaceae SVP‐like TFs are highly variable in the K domain related to oligomerization, which presents a large number of potential positive selection sites (Lai *et al*., 2019; Liu *et al*., 2020). Therefore, evolutionary forces might have contributed not only to the origination of a dormancy‐specific group of TFs but also to increasing their combinatorial complexity in order to modulate the progression of dormancy in the Rosaceae.

### MADS complexes operate during the dormancy cycle of fruit trees

**Fig. 7:**
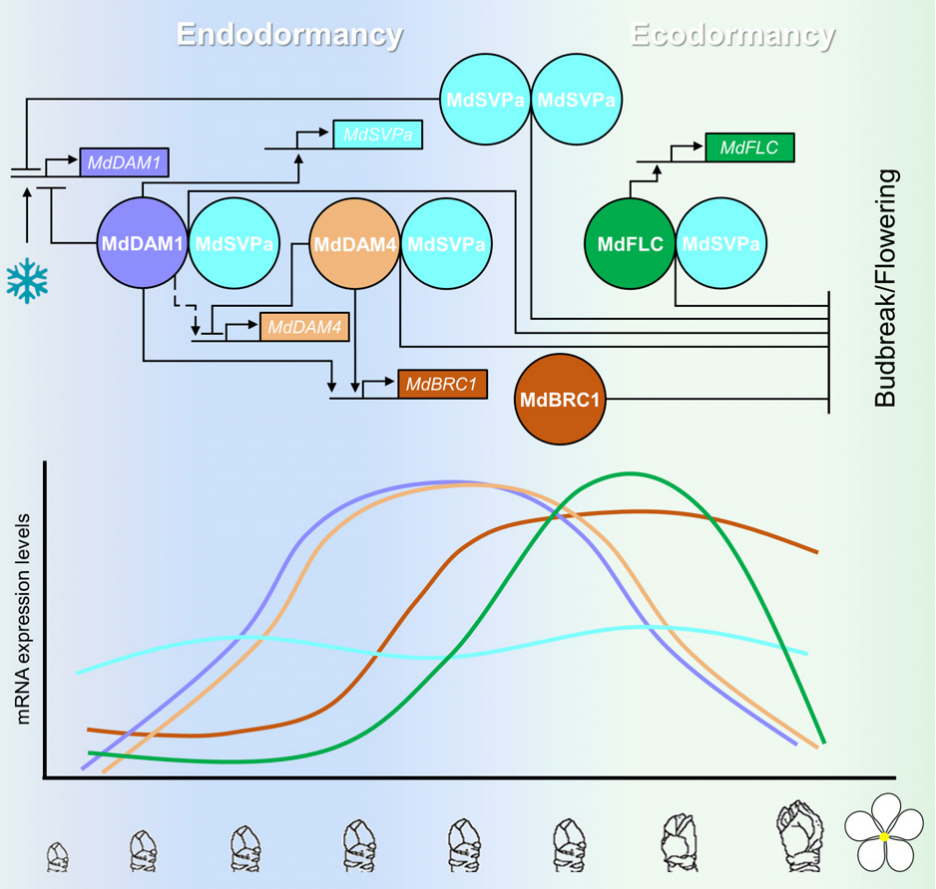
Tentative model summarizing the regulatory interactions between MADS transcription factors (TFs) during the dormancy cycle. Apple MADS TFs MdDAM1, MdDAM4, MdFLC and MdSVPa form complexes and regulate the expression of each other in a genetic regulatory circuit that integrates environmental signals to restrict bud growth during winter dormancy. We propose that this gene regulatory network (GRN) leads to budbreak and flowering inhibition at least partially through the transcriptional control of *MdBRC1*. Lines with arrowheads indicate transcriptional activation, whereas blunted lines represent transcriptional repression. The dotted line represents the induction of *MdDAM4* by MdDAM1 as proposed by Moser *et al*. (2020). Genes and proteins are represented by boxes and circles, respectively. In the lower part of the illustration, the mRNA expression level for each gene is represented (color code as shown in the upper part).

We have demonstrated that the MADS TFs involved in dormancy form molecular complexes, and that MdSVPa is a central component of these complexes (Figs 2a–c, S7a). MdSVPa seems to be crucial for transcriptional function because its absence compromises the ability of the complexes to bind to DNA (Fig. 3a). The relative abundance of these complexes during dormancy might depend on the expression levels of their encoding genes. In Arabidopsis, SVP forms complexes with other MADS TFs to regulate vegetative development, reproductive transition and flower development (de Folter *et al*., 2005). This functional plasticity is partly conferred by the temporal and spatial pattern of expression of *SVP* (Gregis *et al*., 2006; Liu *et al*., 2007; Li *et al*., 2008). Chromatin immunoprecipitation‐sequencing (ChIP‐seq) and transcriptomic experiments showed that switching molecular partners results in distinct SVP DNA‐binding specificity, leading to the expression and/or repression of different sets of target genes (Gregis *et al*., 2013; Mateos *et al*., 2015). Our results indicate that *MdSVPa* is expressed at constant levels during winter, which is compatible with the MdSVPa TF being part of molecular complexes throughout dormancy progression (Figs 2d, S9a,b). Remarkably, *MdDAM1* and *MdDAM4* showed their maximal gene expression during the endodormant phase, whereas *MdFLC* mRNA peaked at the transition from endo‐ to ecodormancy (Fig. 2d). Therefore, we propose a model in which MdSVPa sequentially forms complexes with the MADS TFs that predominate at each dormancy‐cycle phase (Fig. 7).

The recently developed seq‐DAP‐seq technique (Lai *et al*., 2020) has proved to be a powerful tool for determining genome‐wide binding sites of multimeric MADS TFs in fruit trees. Apple MADS combinatory dimerization resulted in a preference for a CArG‐box displaying slightly altered nucleotide composition and A/T core length (Figs 3c, S7b), which could explain differences in target selectivity. Indeed, although many putative target genes were shared between the apple MADS complexes, a significant number of targets were bound by a specific complex (Figs 3e,f, S10). Furthermore, by making use of the GR system (Aoyama & Chua, 1997), we isolated genes that are bound and transcriptionally regulated by the different MADS complexes (Fig. 4). The GO categorization of these target genes evidences the involvement of the apple MADS TFs in several biological processes during the dormancy cycle. Some of these processes are affected by a single MADS TF complex, whereas others are influenced by many of them. These results enable us to conclude that MADS complexes containing MdSVPa are sequentially formed during dormancy to modulate transcriptional responses specific to each dormancy phase (Fig. 7).

### A gene regulatory network governed by SVP‐containing complexes controls the dormancy cycle

We have found significant enrichment of target genes of MdDAM1–MdSVPa, MdDAM4–MdSVPa and MdFLC–MdSVPa complexes within datasets containing dormancy‐related DEGs of apple. Nevertheless, target genes of the MdSVPa–MdSVPa homomeric complex were not statistically significantly enriched in these datasets, suggesting that MdSVPa does not have a main role as a dormancy‐cycle regulator and/or this role requires its association with other MADS TFs. Supporting this idea, the overexpression of *MdSVPa* delays budbreak but does not affect dormancy entrance in apple (Wu *et al*., 2017). Moreover, apple trees in which *MdDAM1* was silenced lost their capacity to enter into dormancy (Moser *et al*., 2020), although *MdSVPa* mRNA is known to be expressed throughout the dormancy cycle in natural conditions (Figs 2d, S9a,b) (Porto *et al*., 2016; Wu *et al*., 2017). In this context, the seasonal regulation of *MdDAM1*, *MdDAM4* and *MdFLC* mRNA levels (Fig. 2d) would impact the composition of the MADS complexes and account for the dynamic expression profiles of their target genes during dormancy. We therefore proposed that apple MADS complexes are potential integrators of temperature signals into GRNs (Fig. 7).

To elucidate these GRNs, we individually classified the target genes of the MdDAM1–MdSVPa, MdDAM4–MdSVPa and MdFLC–MdSVPa complexes in co‐expression clusters during dormancy and analyzed their GO term enrichment (Fig. 6). Several clusters from the three MADS complexes were enriched in categories related to development, reflecting a possible reduction of meristematic activity during endodormancy followed by its reactivation in ecodormancy and at the initiation of budbreak and/or flowering. Remarkably, the MdDAM1–MdSVPa and MdDAM4–MdSVPa target genes related to development have either their maximum or minimum expression levels at the end of endodormancy (i.e. December). This is the precise moment at which the expression levels of *MdDAM1* and *MdDAM4* peak before their rapid downregulation (Fig. S9a). Notably, MdFLC–MdSVPa target genes related to development peaked during ecodormancy (i.e. February), following a similar pattern of expression to that shown by *MdFLC* (Fig. S9a). These results support the notion that MADS complexes directly regulate the transcription of genes related to tree developmental processes during dormancy. Moreover, the MdDAM1–MdSVPa complex inhibits the expression of a set of genes related to ‘growth’ and ‘cell growth’ at the moment of the highest expression level of *MdDAM1* (Fig. 6a, cluster 3). This suggests a key role of the MdDAM1–MdSVPa complex in growth repression during endodormancy, which is supported by the evergrowing‐like phenotype displayed by transgenic apple trees silencing *MdDAM1* mRNA expression (Moser *et al*., 2020). Besides growth and development, numerous GO categories associated with response to stress and hormones, cell wall modifications, carbohydrate metabolism, and signaling were enriched in the clusters summarized in Fig. 6 and Dataset S9, and their association to the dormancy cycle has been reported elsewhere (Fadón *et al*., 2020).

A list of 231 MADS complexes’ target genes with potential function during dormancy was produced (Dataset S11), which included homologues of Arabidopsis genes playing roles in bud and meristem development (*BRC1* and *MP*), hormone homeostasis and signaling (*CKX5*, *GA2ox1*, *NCED4* and *PYL4*), cell wall remodeling (*XTH9, XTH15* and *XTH23*) and flowering‐time control (*SOC1*, *LHY* and *CDF2*). Notably, homologues of the Arabidopsis *BRC1* are known to act as bud outgrowth repressors in several plant species (Aguilar‐Martínez *et al*., 2007; Wang *et al*., 2019; Vayssières *et al*., 2020). In hybrid aspen, SVP‐like (SVL) operates upstream of *BRC1* and the GA and ABA signaling cascades. The direct binding of SVL to the *BRC1* locus induces its transcriptional activation, and *BRC1* overexpression leads to late budbreak. In turn, the expression of *SVL* and *BRC1* is downregulated by cold, explaining, at least in part, a mechanism of temperature‐mediated regulation of budbreak in trees (Singh *et al*., 2018). Here, we found that MdDAM1–MdSVPa and MdDAM4–MdSVPa complexes bind to *MdBRC1* and induce its mRNA expression (Fig. S10a). Furthermore, *MdBRC1* expression (Fig. 5c, cluster 16) is induced at the moment when *MdDAM1* and *MdDAM4* exhibit their highest expression levels (Fig. S9a). Similar gene expression dynamics during dormancy were reported for *MdBRC1* (Wu *et al*., 2021). However, this dynamic was not disrupted in evergrowing‐like RNAi plants simultaneously targeting several apple *DAM*‐ and *SVP*‐like genes, suggesting that other pathways also contribute to *MdBRC1* transcriptional modulation. Although the role of MdBRC1 role has not yet been proven in apple trees, it is very likely that it acts as a budbreak repressor, as reported in most of the plant species in which it has been studied so far (Aguilar‐Martínez *et al*., 2007; Wang *et al*., 2019; Vayssières *et al*., 2020). Thus, we propose that MdBRC1 functions downstream of MdSVPa‐containing complexes to repress budbreak during ecodormancy, probably together with the MdFLC–MdSVPa complex.

The GRN described here could also be partially regulated by feedback loops between MADS complexes. This tightly controlled regulation is likely necessary to orchestrate the dormancy‐cycle progression. MdSVPa, which does not exhibit significant changes in expression during dormancy, acts as an organizing hub for DAM‐ and FLC‐like MADS TF complexes, whose expression patterns do change during the dormancy cycle. These heteromeric complexes are required for expression of distinct sets of target genes. Taken together, our results show that apple DAM‐, FLC‐ and SVP‐like complexes operate in different GRNs that integrate environmental and hormonal signaling pathways to regulate dormancy (Fig. 7).

## Author contributions

VdSF, ES, XL, JE, IF and VH performed the experiments, VSF and ES analyzed the data, VdSF, LFR, CZ, GC, EC and FA conceived and designed the experiments, VdSF, ES and FA wrote the manuscript. All the authors approved the final version.

## Supporting information


**Dataset S1** List of seq‐DAP‐seq peaks for the four MADS transcriptional complexes.

**Dataset S2** List of seq‐DAP‐seq peaks containing at least one CArG‐box (see the Materials and Methods section) for the four MADS transcriptional complexes.

**Dataset S3** RNA‐seq result of the glucocorticoid receptor (GR) DEX‐inducible assay in apple calli for four MADS TFs.

**Dataset S4** List of high‐confidence targets of four MADS transcriptional complexes.

**Dataset S5** Heatmap summarizing the gene expression during dormancy of the high‐confidence targets of the MdDAM1–MdSVPa, MdDAM4–MdSVPa and MdFLC–MdSVPa complexes.

**Dataset S6** Heatmap summarizing the gene expression during dormancy of the high‐confidence targets of the MdDAM1–MdSVPa complex.

**Dataset S7** Heatmap summarizing the gene expression during dormancy of the high‐confidence targets of the MdDAM4–MdSVPa complex.

**Dataset S8** Heatmap summarizing the gene expression during dormancy of the high‐confidence targets of the MdFLC–MdSVPa complex.

**Dataset S9** Enrichment analysis of GO terms for biological processes over the clusters formed during dormancy for the target genes of the MdDAM1–MdSVPa, MdDAM4–MdSVPa, and MdFLC–MdSVPa complexes.

**Dataset S10** List of genes that are differentially regulated during dormancy in dataset A and dataset B.

**Dataset S11** List containing 231 dormancy‐related DEGs that are high‐confidence targets of the MdDAM1–MdSVPa, MdDAM4–MdSVPa, and/or MdFLC–MdSVPa complexes.

**Fig. S1** Flowering‐time distributions shown as leaf number at bolting.
**Fig. S2** Expression pattern of *pSVP*::*Venus* lines.
**Fig. S3** Protein–protein interactions among apple DAM‐, SVP‐ and FLC‐like proteins.
**Fig. S4** Subcellular localization of apple DAM‐, SVP‐ and FLC‐like proteins.
**Fig. S5** Flowering time under LD of Arabidopsis F1 plants in comparison to wild‐type and homozygous lines.
**Fig. S6** Chilling hours accumulated during the dormancy cycle.
**Fig. S7** Additional information about the seq‐DAP‐seq assays.
**Fig. S8** Additional information about the GR DEX‐inducible assays.
**Fig. S9** Expression analysis of *MdDAM1*, *MdDAM4*, *MdFLC* and *MdSVPa* in two public RNA‐seq datasets during apple dormancy.
**Fig. S10** MADS transcriptional complexes regulate several dormancy‐related genes.
**Methods S1** Supporting methods.
**Table S1** List of primers used in this work.
Please note: Wiley Blackwell are not responsible for the content or functionality of any Supporting Information supplied by the authors. Any queries (other than missing material) should be directed to the *New Phytologist* Central Office.

## Data Availability

The datasets generated during the current study are available in the NCBI SRA repository (PRJNA698061).
