# Supplementary material for: Unraveling the role of MADS transcription factor complexes in apple tree dormancy
Source: New Phytol. 2021 Sep 23;232(5):2071–88. doi: 10.1111/nph.17710 (PMC9292984; doi:10.1111/nph.17710)
Supplement: Supplementary file 6 — Dataset S6 Heatmap summarizing the gene expression during dormancy of the high‐confidence targets of the MdDAM1–MdSVPa complex. [file NPH-232-2071-s007.pdf]

New Phytologist

New Phytologist Supporting Information

Article title: Unraveling the role of MADS transcription factor complexes in apple tree dormancy

Authors: Vítor da Silveira Falavigna, Edouard Severing, Xuelei Lai, Joan Estevan, Isabelle Farrera, Véronique Hugouvieux, Luís Fernando Revers, Chloe Zubieta, George Coupland, Evelyne Costes, Fernando Andrés

Article acceptance date: 19 August 2021

Data S6. Heatmap summarizing the gene expression during dormancy of the high-confidence targets of MdDAM1–MdSVPa complex. The Z-score expression values corresponding to the colors in the heatmap are indicated by the colormap. The histogram in the colormap indicates the frequencies of each color in the heatmap.

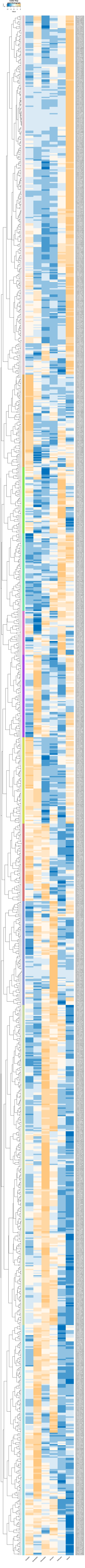
Color Key
and Histogram
Count
0
−2
−1
0
1
2
Value
