# Supplementary material for: Unraveling the role of MADS transcription factor complexes in apple tree dormancy
Source: New Phytol. 2021 Sep 23;232(5):2071–88. doi: 10.1111/nph.17710 (PMC9292984; doi:10.1111/nph.17710)
Supplement: Supplementary file 8 — Dataset S8 Heatmap summarizing the gene expression during dormancy of the high‐confidence targets of the MdFLC–MdSVPa complex. [file NPH-232-2071-s009.pdf]

New Phytologist

***New Phytologist* Supporting Information**

Article title: Unraveling the role of MADS transcription factor complexes in apple tree dormancy

Authors: Vítor da Silveira Falavigna, Edouard Severing, Xuelei Lai, Joan Estevan, Isabelle Farrera, Véronique Hugouvieux, Luís Fernando Revers, Chloe Zubieta, George Coupland, Evelyne Costes, Fernando Andrés

Article acceptance date: 19 August 2021

Data S8. Heatmap summarizing the gene expression during dormancy of the high-confidence targets of MdFLC–MdSVPa complex. The Z-score expression values corresponding to the colors in the heatmap are indicated by the colormap. The histogram in the colormap indicates the frequencies of each color in the heatmap.

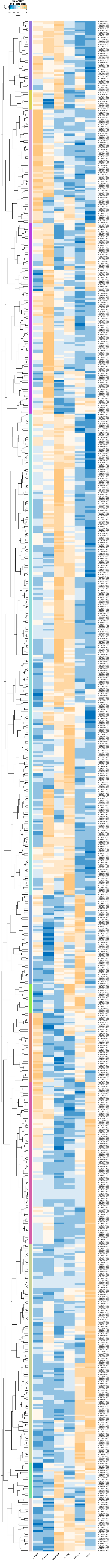
Color Key
and Histogram
Count
0
−2
−1
0
1
2
Value
October
November
December
January
February
March
