# Supplementary material for: Unraveling the role of MADS transcription factor complexes in apple tree dormancy
Source: New Phytol. 2021 Sep 23;232(5):2071–88. doi: 10.1111/nph.17710 (PMC9292984; doi:10.1111/nph.17710)
Supplement: Supplementary file 12 — Fig. S1 Flowering‐time distributions shown as leaf number at bolting. Fig. S2 Expression pattern of pSVP::Venus lines. Fig. S3 Protein–protein interactions among apple DAM‐, SVP‐ and FLC‐like proteins. Fig. S4 Subcellular localization of apple DAM‐, SVP‐ and FLC‐like proteins. Fig. S5 Flowering time under LD of Arabidopsis F1 plants in comparison to wild‐type and homozygous lines. Fig. S6 Chilling hours accumulated during the dormancy cycle. Fig. S7 Additional information about the seq‐DAP‐seq assays. Fig. S8 Additional information about the GR DEX‐inducible assays. Fig. S9 Expression analysis of MdDAM1, MdDAM4, MdFLC and MdSVPa in two public RNA‐seq datasets during apple dormancy. Fig. S10 MADS transcriptional complexes regulate several dormancy‐related genes. Methods S1 Supporting methods. Table S1 List of primers used in this work. Please note: Wiley Blackwell are not responsible for the content or functionality of any Supporting Information supplied by the authors. Any queries (other than missing material) should be directed to the New Phytologist Central Office. [file NPH-232-2071-s005.pdf]

New Phytologist **Supporting Information**

Article title: Unraveling the role of MADS transcription factor complexes in apple tree dormancy
Authors: Vítor da Silveira Falavigna, Edouard Severing, Xuelei Lai, Joan Estevan, Isabelle Farrera, Véronique Hugouvieux, Luís Fernando Revers, Chloe Zubieta, George Coupland, Evelyne Costes, Fernando Andrés
Article acceptance date: 19 August 2021

The following Supporting Information is available for this article:

Fig. S1 Flowering-time distributions shown as leaf number at bolting. a) T1 populations transformed with the different apple *DAM*-like, *SVP*-like or control genes. X-axis shows rosette numbers of leaves of individual plants, and y-axis shows the numbers of individual plants. The red square represents the median for each gene population. **b)** Total leaf number (including cauline and rosette leaves) at bolting. The box extends from the 25th to 75th percentiles, the line in the middle is plotted at the median, and the whiskers are drawn down to the 10th and up to the 90th percentile. The outliers below and above the whiskers are drawn as individual points. Kruskal-Wallis one-way ANOVA followed by Dunn's test was used for the statistical tests. Letters shared in common between the genotypes indicate no significant differences (for P ≤ 0.05). Plants representing intermediate phenotypes of each genotype grown under LDs for 21 days.

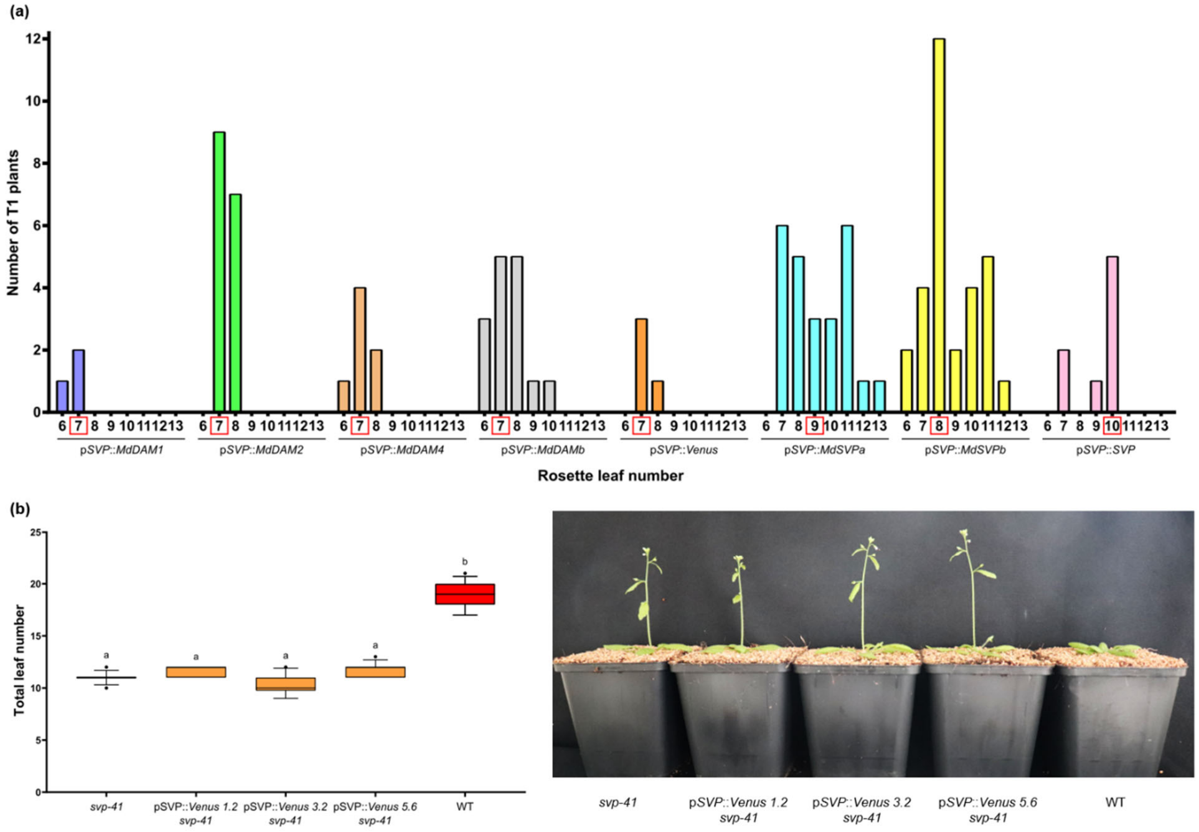

Fig. S2 Expression pattern of *pSVP*::*Venus* lines. Confocal analysis of expanded leaves (7-day-old plants) or dissected apices (10-day-old plants) of *pSVP*::*Venus* lines.

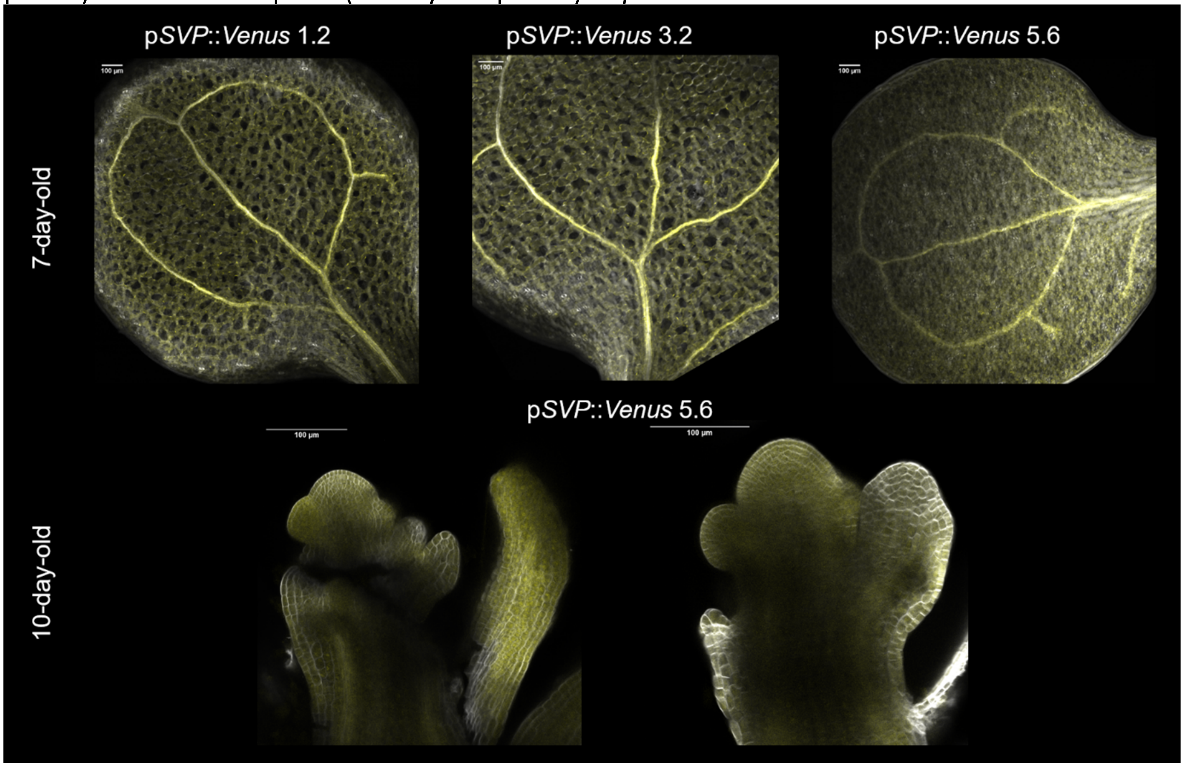

Fig. S3 Protein–protein interactions among apple DAM-, SVP- and FLC-like proteins. a) Yeast two-hybrid assay using full-length apple proteins. MdDAM1, MdDAM2 and MdDAM4 showed strong autoactivation even in the drop-out media supplemented with 5 mM of 3AT. To circumvent this issue, the C-terminal region of all proteins was removed, as it is known that this region is responsible for the generation of autoactivation in MADS-domain proteins. **b)** Yeast two-hybrid assays using truncated protein versions. Interactions were tested in both directions, unless a positive interaction was identified before. In this case, the other direction was not tested (blank spaces with nt – not tested). Proteins that did not produce autoactivation were not evaluated in the drop-out media supplemented with 3AT. The negative controls are representative pictures of several independent controls that were used in this assay.

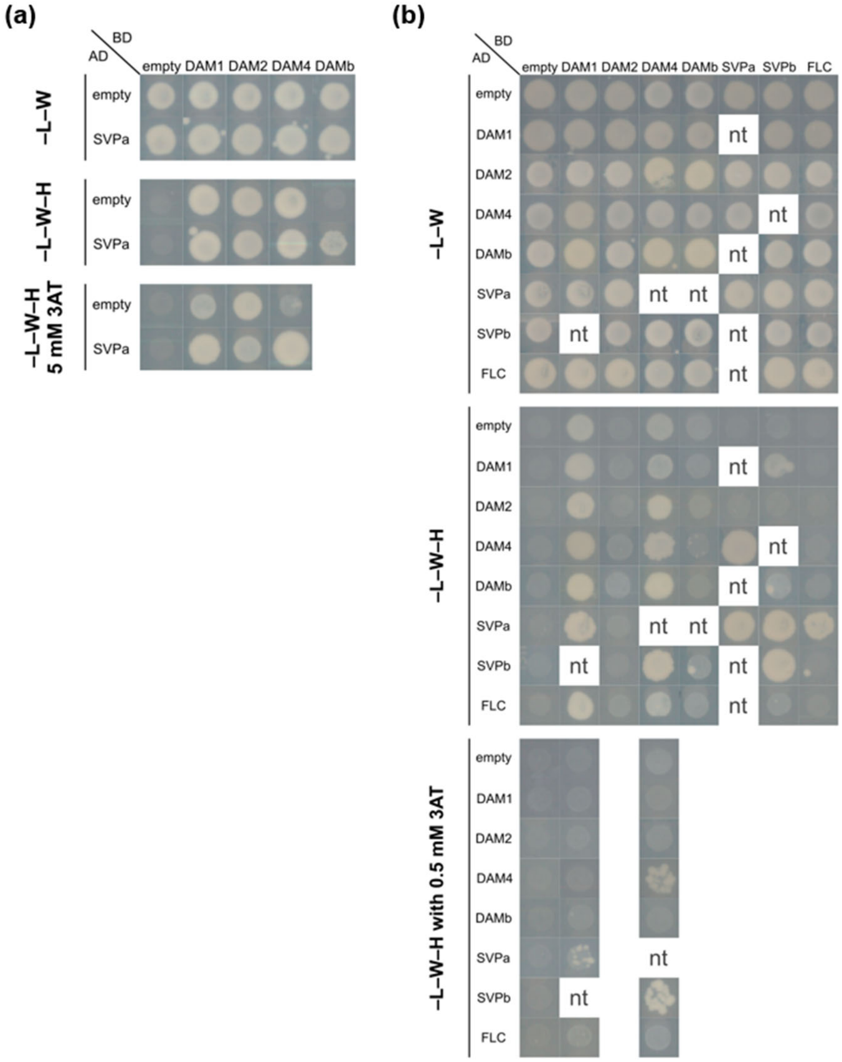

Fig. S4 Subcellular localization of apple DAM-, SVP- and FLC-like proteins. MdDAM1 **(a)**, MdDAM2 **(b)**, MdDAM4 **(c)**, MdDAMb **(d)**, MdSVPa **(e)**, MdSVPb **(f)** and MdFLC **(g)** were translationally fused to GFP and *Nicotiana benthamiana* leaves were agroinfiltrated and observed in a confocal microscope after 3 days of incubation. **h)** VENUS fused to a nuclear localization signal (NLS). Scale bar 100 µm.

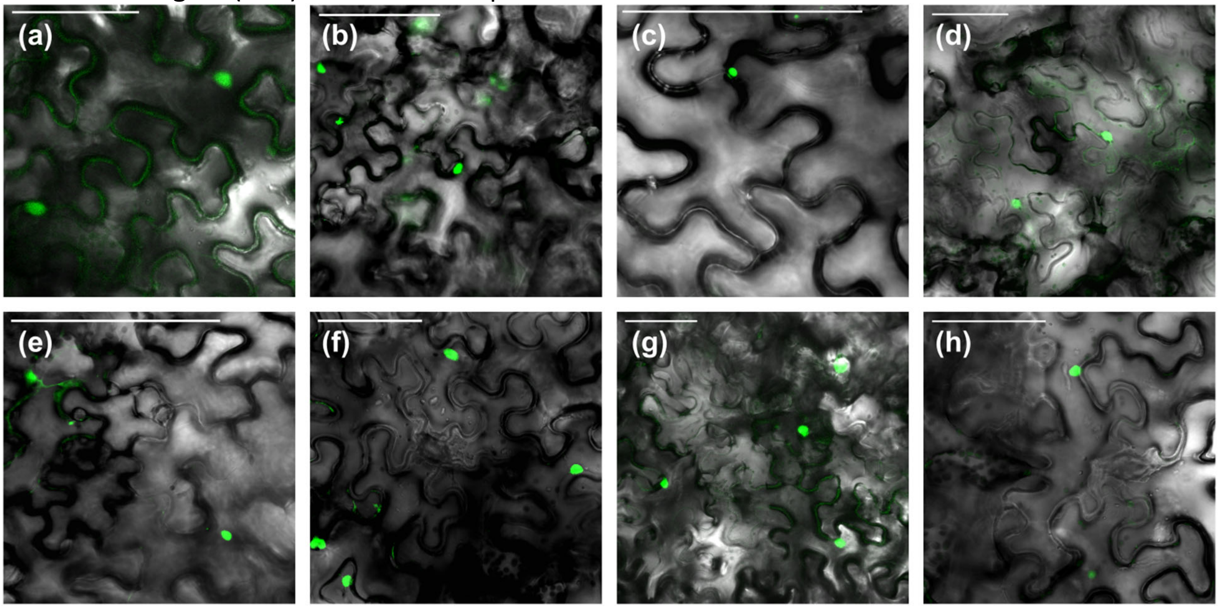

Fig. S5 Flowering time under LD of Arabidopsis F1 plants in comparison to WT and homozygous lines. a) Total leaf number (including both cauline and rosette leaves) was scored prior to flowering. **b)** Number of days from germination to bolting (elongation of the first internode around 0.5 cm). **c)** Number of days from germination to the opening of the first flower. The box extends from the 25th to 75th percentiles, the line in the middle is plotted at the median, and the whiskers are drawn down to the 10th and up to the 90th percentile. The outliers below and above the whiskers are drawn as individual points. One-way ANOVA followed by Tukey's test was used for the statistical tests. Letters shared in common between the genotypes indicate no significant differences (for P ≤ 0.05).

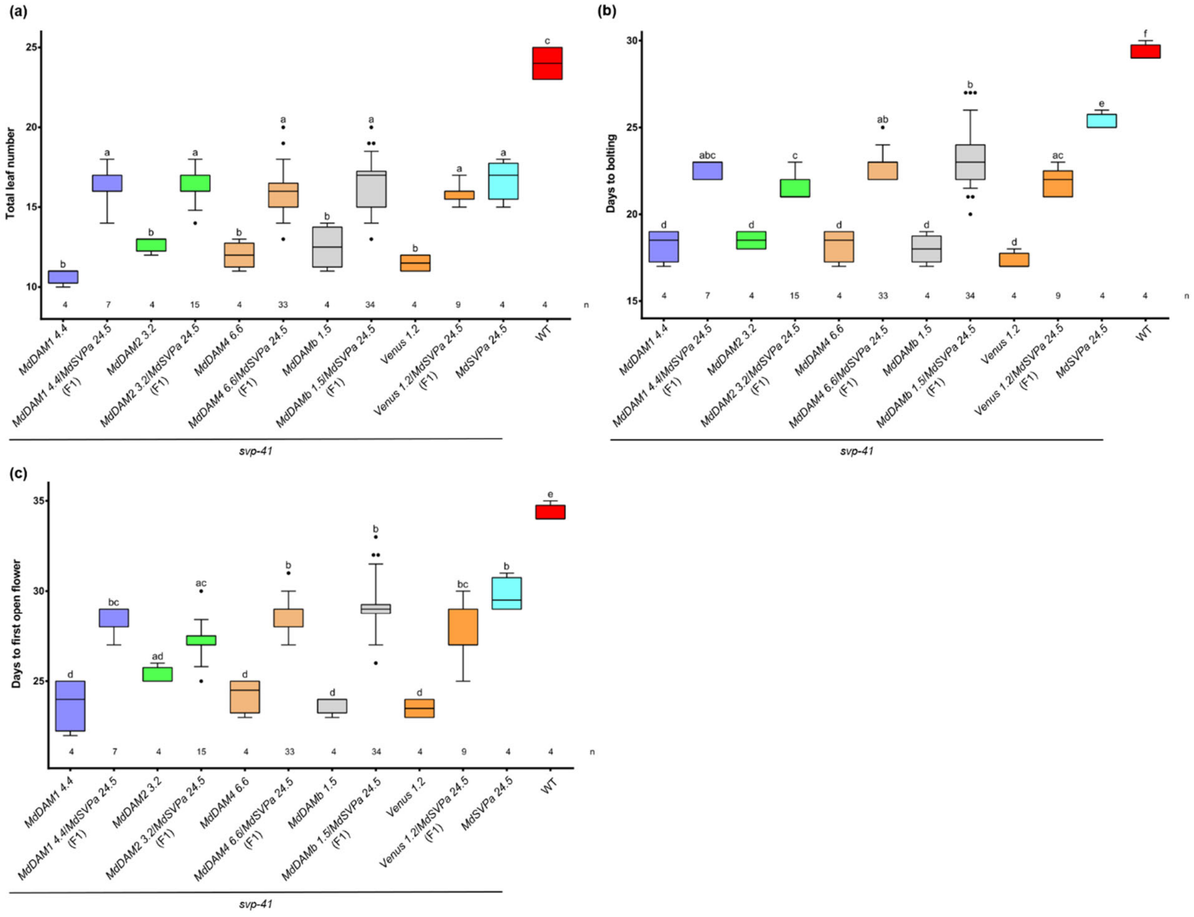

Fig. S6 Chilling hours accumulated during the dormancy cycle. The accumulation of chilling hours (number of hours with temperatures below 7.2 °C) was monitored by daily recording the air temperature in field trials. Red arrows represent sampling points for RT-qPCR studies.

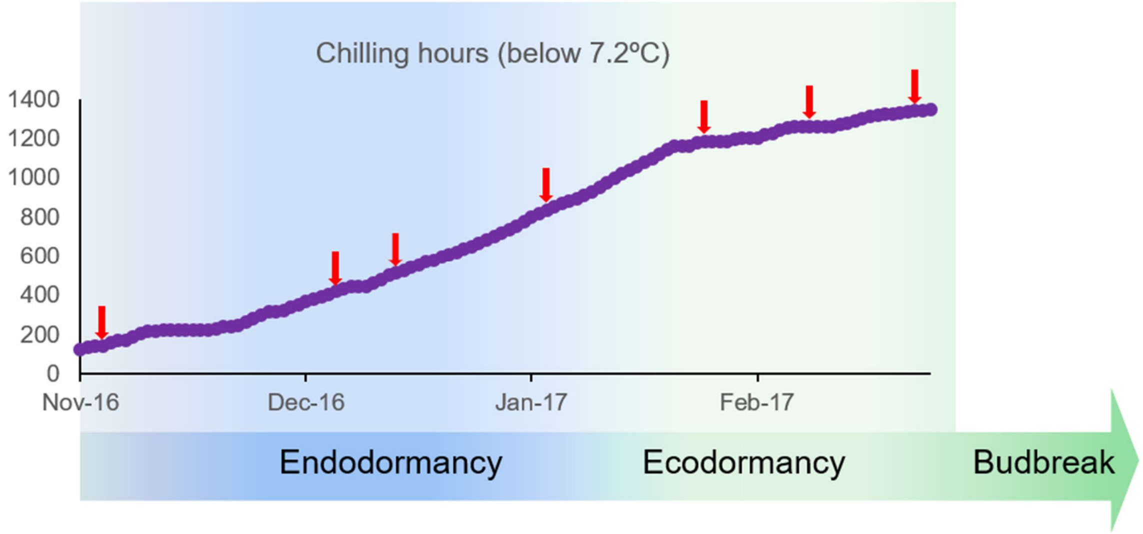

Fig. S7 Complementary information about the seq-DAP-seq assays. a) *In vitro* co-immunoprecipitation of proteins. MdDAM1, MdDAM4, MdFLC and MdSVPa were translationally fused to FLAG, whereas MdDAM1, MdDAM4, MdDAMb, MdFLC and MdSVPa were translationally fused to MyC. Protein fusions were synthesized *in vitro* and tested in pairs. The input was composed of total proteins recovered before immunoprecipitation. FLAG-fused proteins were immunoprecipitated using anti-FLAG beads and immunoblotted using anti-FLAG or anti-MyC antibody. **b)** Logos of the enriched CArG-box motifs identified with the degenerated CArG-box strategy (see Methods S1). **c)** Frequency distribution of CArG-box motifs in peaks associated to genes for each complex and to 1,000 random sets. Bars represent 95% confidence interval. **d)** GO term enrichment analysis of target genes containing a CArG-box for each apple transcriptional complex. For data visualization, the best P-value was transformed using log. Note that all P-values higher than 0.05 were replaced by zero (white boxes). **e)** DNA-binding profiles of the four complexes and the control (input) to the locus region of *MdDAM1*, *MdDAMb* and *MdFLC*. Horizontal bars below the plots represent the position of the peak regions. The color code between the plots and the bars is preserved. The Integrated Genome Browser (IGB) was used for visualization.

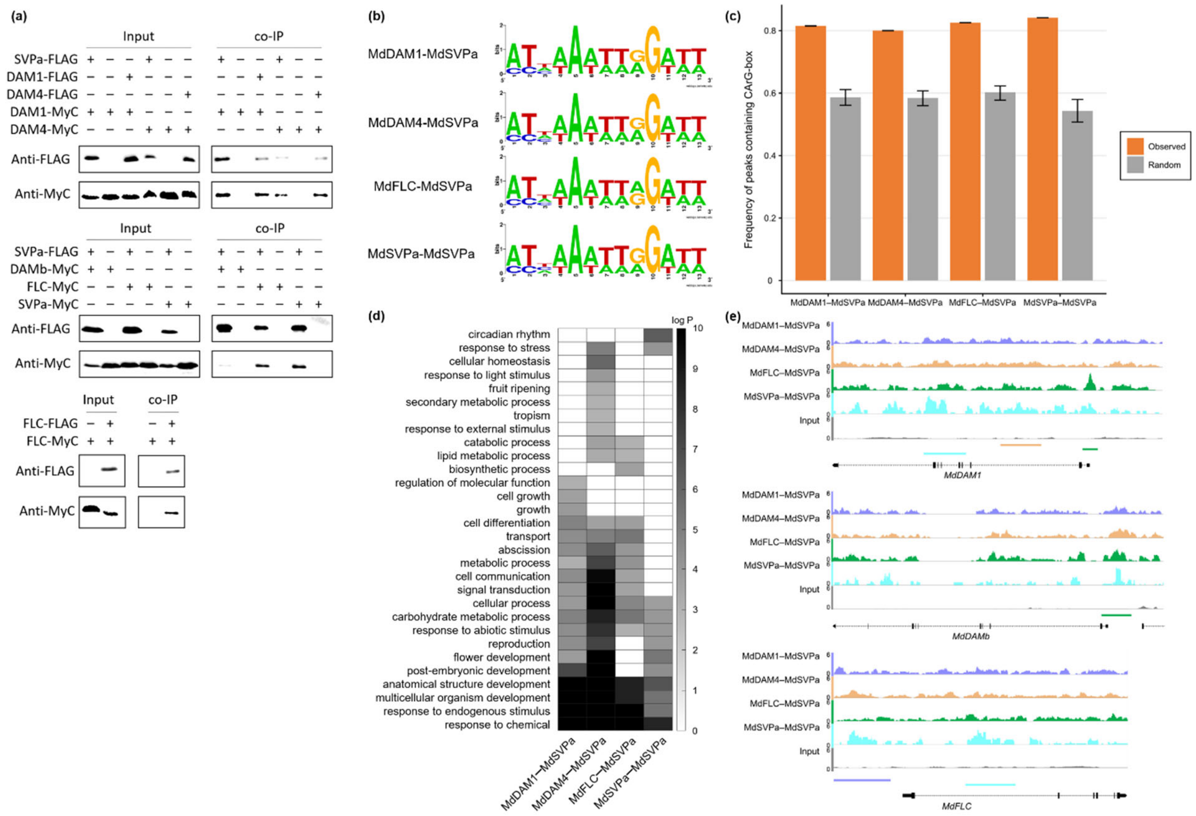

Fig. S8 Complementary information about the GR DEX-inducible assays. a) Dendrogram illustrating the distribution of the RNA-seq replicates in the GR DEX-inducible assay performed in transgenic apple calli. **b)** Venn diagram illustrating common DEGs between the four TFs using the GR DEX-inducible system. **c)** GO term enrichment analysis of genes identified in transgenic apple calli after 8 hours of DEX induction. Enrichment tests were performed separately for up- and downregulated gene sets and only the best P-value (smallest) was kept. For data visualization, the best P-value was transformed using -log or log when it belonged to the up (orange gradient) or downregulated (blue gradient) gene set, respectively. Note that all P-values higher than 0.05 were replaced by zero (white boxes).

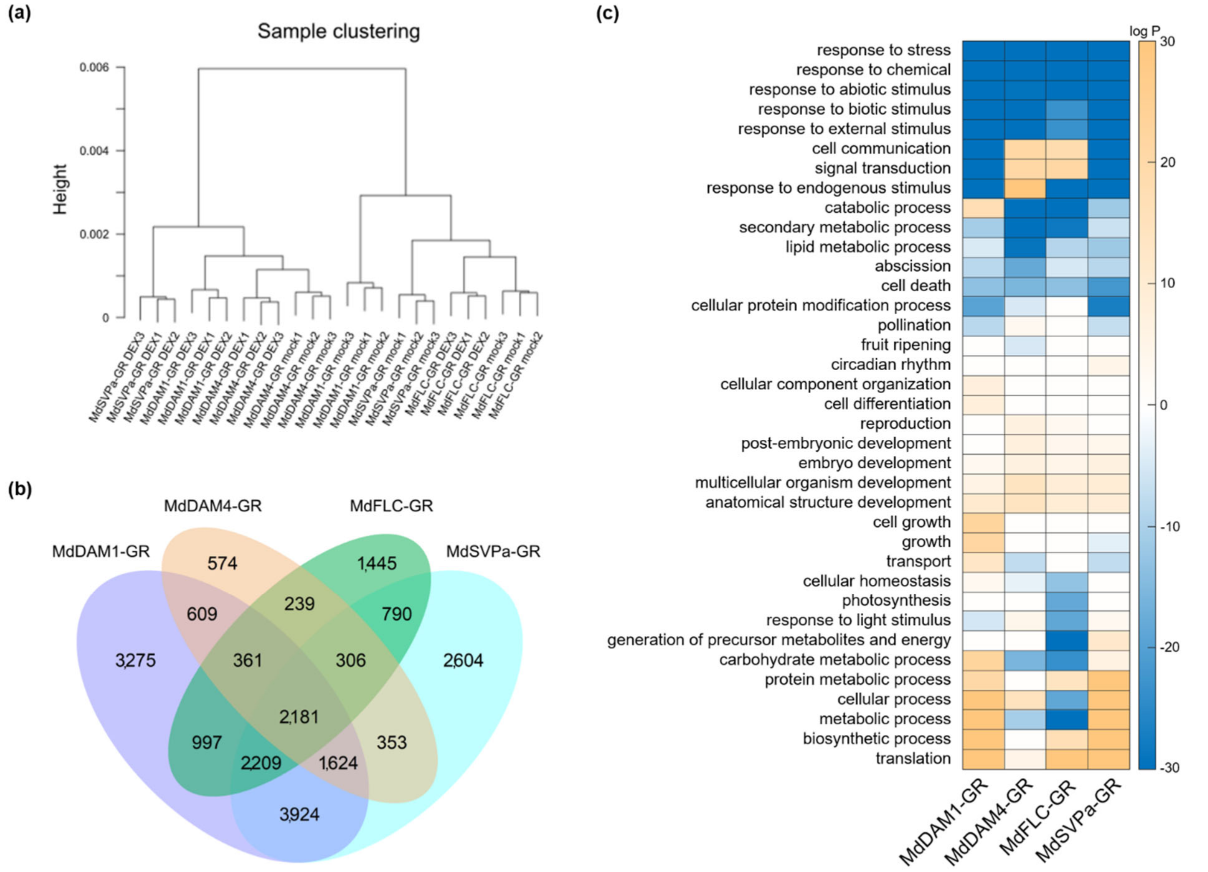

Fig. S9 Expression analysis of *MdDAM1*, *MdDAM4*, *MdFLC* and *MdSVPa* in two public RNA-seq during apple dormancy. a) Time-course expression analysis of apple buds harvested from field-grown 'Golden delicious' trees according to Moser et al. 2020. **b)** Expression analysis of apple bud samples harvested from 'Fuji' trees in the field and exposed to controlled chilling conditions according to Takeuchi et al. 2018. In **(a)** and **(b)**, bars represent the minimum and maximum values obtained. **c)** Venn diagram illustrating the overlap between dormancy-related DEGs. Only genes present in both datasets were considered in this analysis. The obtained P-value (hypergeometric test) for the intersection between datasets was equal to 0.

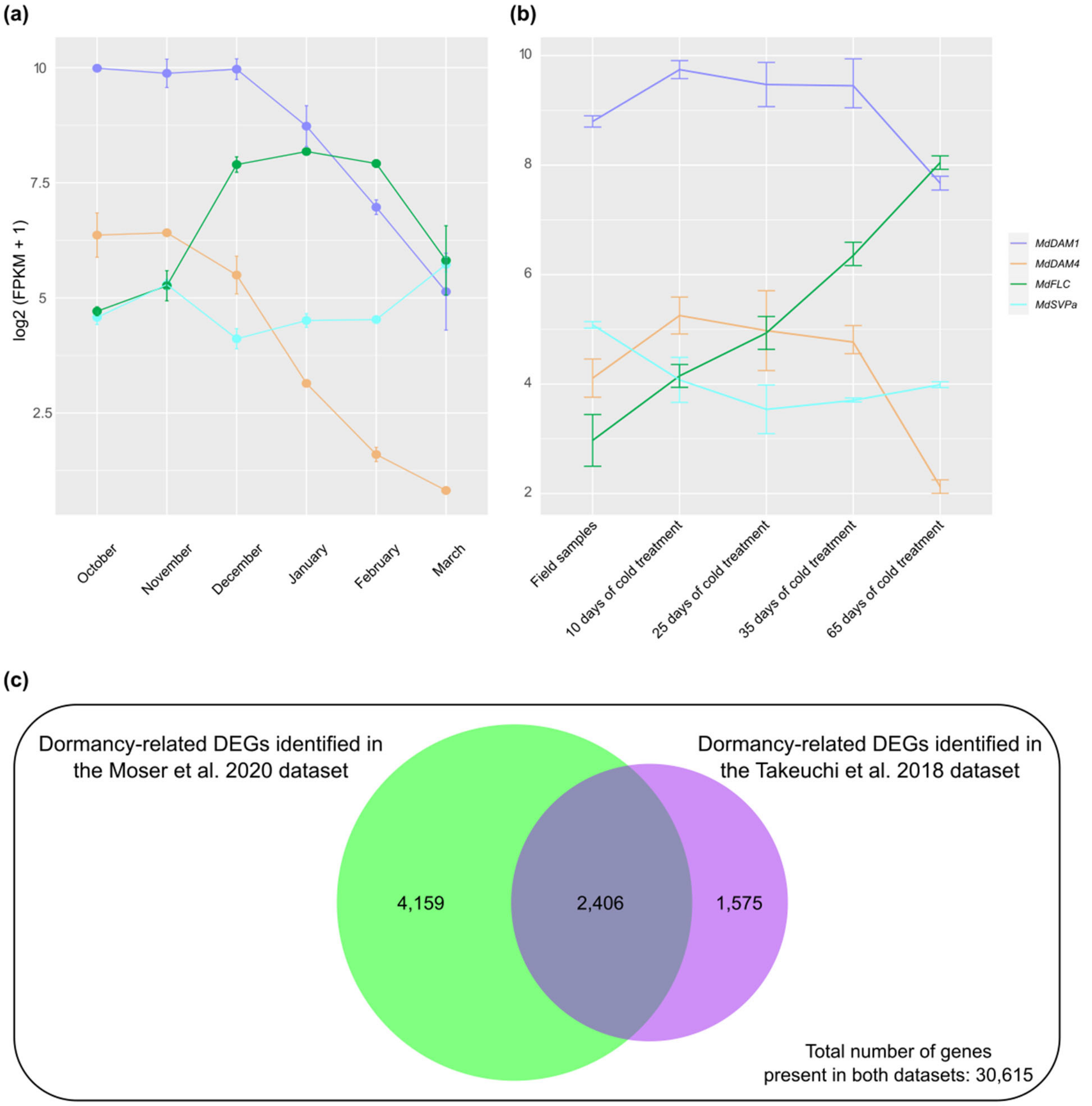

Fig. S10 MADS transcriptional complexes regulate several dormancy-related genes. a) DNA-binding profiles of four MADS transcriptional complexes and the control (input) to the locus region of several dormancy-related genes. **b)** DNA-binding profiles of four MADS transcriptional complexes and the control (input) to the locus region of *MdSOC1a*. Time-course expression analysis of apple buds harvested from field-grown 'Golden delicious' trees according to Moser et al. 2020. Bars represent the minimum and maximum values obtained. The Integrated Genome Browser (IGB) was used for visualization. The arrows represent induction or repression according to the expression data obtained in the calli assay for genes belonging to the high-confidence list of target genes (Data **S4**).

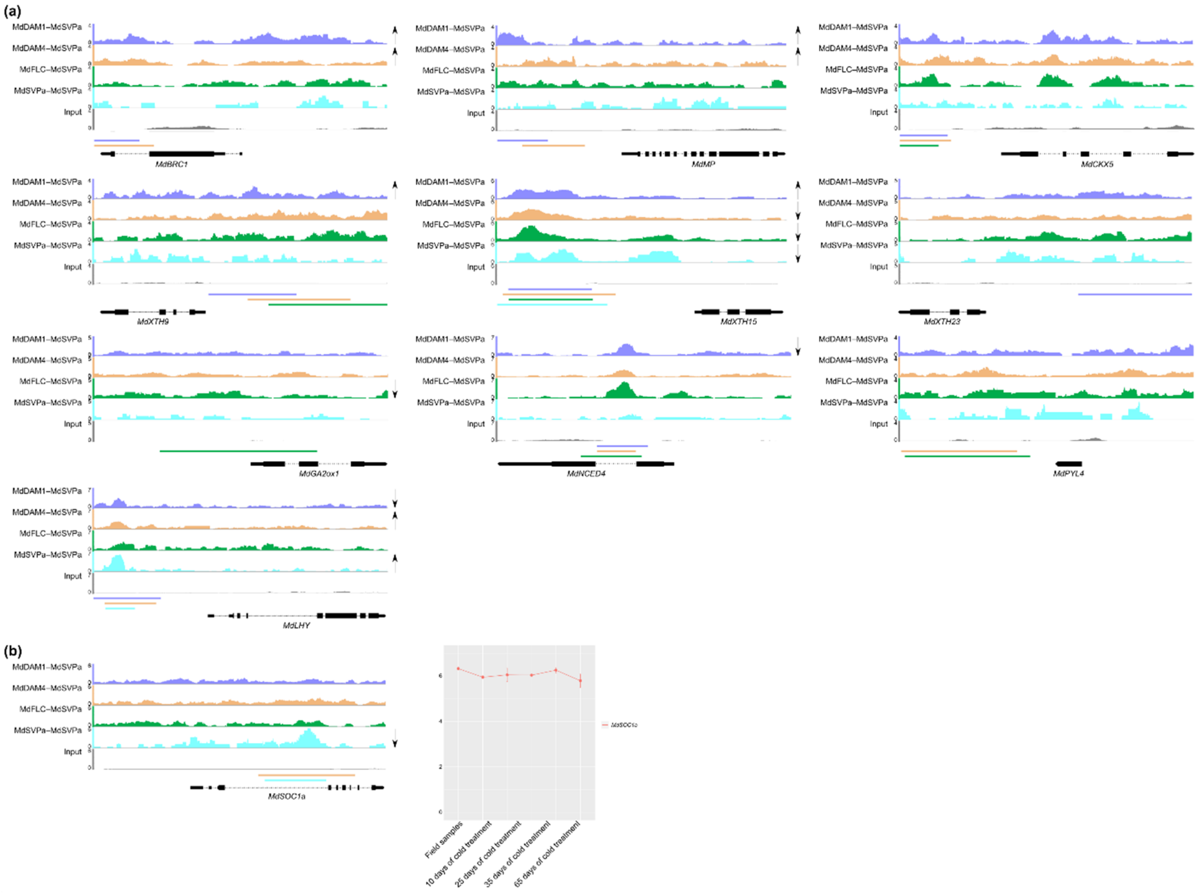

New Phytologist

Table S1 List of primers employed in this work.

Purpose	Primer name	Accession code	Sequence (5' -> 3')	Reference
Expression analysis	MdDAM1-F3	MD15G1384500	GGAGAAGGGGGATTTTCAAGA	Porto *et al.*, 2016
	MdDAM1-R3	MD15G1384500	CGTGATGAATCGTGGGTTGA	Porto *et al.*, 2016
	MdDAM2-F3	MD08G1196900	TGAAGAGCTGTCGATTCTGTGTG	Porto *et al.*, 2016
	MdDAM2-R3	MD08G1196900	CGATTTTTCCCCAGTATGTGA	Porto *et al.*, 2016
	MdDAM4-F3	MD08G1197300	ATATGAAGGGTGAGGACCT	Porto *et al.*, 2016
	MdDAM4-R3	MD08G1197300	CGTAAGTGCCATAATCTCAC	Porto *et al.*, 2016
	MdDAMb-F	MD15G1384600	AGGATGGTGATGTTATCCGGA	Wu *et al.*, 2017
	MdDAMb-R	MD15G1384600	CTAAGGAAGCCCGAGTTTGAGA	This work
	MdSVPa-RT-F	MD01G1038600	TACACCAGGTTGAGCAAGGA	This work
	MdSVPa-RT-R	MD01G1038600	CATCAGATTTCTCCGCCACT	This work
	MdSVPb-RT-F	MD15G1313200	TGCATTCAAAGAATCTCGACAA	Porto *et al.*, 2016
	MdSVPb-RT-R	MD15G1313200	TTCCTTGCTCAACCTGGAGTA	This work
	MdFLC-like-F	MD09G1009100	AACAGATGAAAGAAGAGAAGGTTCG	Porto *et al.*, 2015
	MdFLC-like-R	MD09G1009100	TATTAGCGGCGGAAGTGCTC	Porto *et al.*, 2015
	MdMDH-F	MD16G1219000	CGTGATTGGGTACTTGGAAC	Perini *et al.*, 2014
	MdMDH-R	MD16G1219000	TGGCAAGTGACTGGGAATGA	Perini *et al.*, 2014
	MdWD40-F	MD08G1215900	GGATTTACTGTGTTGGTGAAG	Perini *et al.*, 2014
	MdWD40-R	MD08G1215900	TGCCAATTACCTCCTTTTCGTG	Perini *et al.*, 2014
	SVP-RT-F	AT2G22540	GAAGGACAGTCGTCGGAGTC	Mateos *et al.*, 2015
	NOS-T-R	-	GCAAGACCGGCAACAGGATT	This work
	Venus-RT-F	-	CGACCACTACCAGCAGAACA	This work
	Venus-RT-R	-	GAACTCCAGCAGGACCATGT	This work
	PPA2-RT-F	AT1G13320	CAGCAACGAATTGTGTTTGG	Mateos *et al.*, 2015
	PPA2-RT-R	AT1G13320	AAATACGCCCAACGAACAAA	Mateos *et al.*, 2015
Gateway cloning	MdDAM1-BP-F	MD15G1384500	GGGGACAAGTTTGTACAAAAAAGCAGGCTCTATGAAGATCAAGATAAAAAAG	This work
	MdDAM1-BP-R	MD15G1384500	GGGGACCACTTTGTACAAGAAAGCTGGGTCTACGGAAGCCCCAGTTTG	This work
	MdDAM1-trunc-BP-R	MD15G1384500	GGGGACCACTTTGTACAAGAAAGCTGGGTCTATGATTCAGATGTCACGCTTTC	This work
	MdDAM1-noStop-BP-R	MD15G1384500	GGGGACCACTTTGTACAAGAAAGCTGGGTtCGGAAGCCCCAGTTTG	This work
	MdDAM2-BP-F	MD08G1196900	GGGGACAAGTTTGTACAAAAAAGCAGGCTCTATGGTGAAAATAAGGAAAGAG	This work
	MdDAM2-BP-R	MD08G1196900	GGGGACCACTTTGTACAAGAAAGCTGGGTCTAAGGTAGCCCCAGTTTGAGA	This work
	MdDAM2-trunc-BP-R	MD08G1196900	GGGGACCACTTTGTACAAGAAAGCTGGGTCTAATTTGTAGCTGATTCAGATGTC	This work
	MdDAM4-BP-F	MD08G1197300	GGGGACAAGTTTGTACAAAAAAGCAGGCTCTATGGTGAAAAGGATGAATGAGAAG	This work
	MdDAM4-BP-R	MD08G1197300	GGGGACCACTTTGTACAAGAAAGCTGGGTCTAAGGAAGCCCCAGTTTGAGA	This work
	MdDAM4-trunc-BP-R	MD08G1197300	GGGGACCACTTTGTACAAGAAAGCTGGGTCTAGACATTTGTGGCTGATTCAGA	This work
	MdDAM4-noStop-BP-R	MD08G1197300	GGGGACCACTTTGTACAAGAAAGCTGGGTtAGGAAGCCCCAGTTTGAGA	This work
	MdDAMb-BP-F	MD15G1384600	GGGGACAAGTTTGTACAAAAAAGCAGGCTCTATGATGAGGGAGAAGATACAGATC AAGAA	This work
	MdDAMb-BP-R	MD15G1384600	GGGGACCACTTTGTACAAGAAAGCTGGGTCTAAGGAAGCCCGAGTTTGAGA	This work

	MdDAMb-trunc-BP-R	MD15G1384600	GGGGACCACTTTGTACAAGAAAGCTGGGTTCACGCAGGTCCGATATCTCCTC	This work
	MdSVPa-BP-F	MD01G1038600	GGGGACAAGTTTGTACAAAAAAGCAGGCTCTATGGCGAGGGAGAAAATTCAG	This work
	MdSVPa-BP-R	MD01G1038600	GGGGACCACTTTGTACAAGAAAGCTGGGTTCAACCAGAGTAAGGCAGCC	This work
	MdSVPa-trunc-BP-R	MD01G1038600	GGGGACCACTTTGTACAAGAAAGCTGGGTCTAGCCATCAGATTTCTCCGCCAC	This work
	MdSVPa-noStop-BP-R	MD01G1038600	GGGGACCACTTTGTACAAGAAAGCTGGGTtACCAGAGTAAGGCAGCC	This work
	MdSVPb-BP-F	MD15G1313200	GGGGACAAGTTTGTACAAAAAAGCAGGCTCTATGGCGAGGGAGAAGATTCAG	This work
	MdSVPb-BP-R	MD15G1313200	GGGGACCACTTTGTACAAGAAAGCTGGGTTTAAGCGCACCCCAATTTTAG	This work
	MdSVPb-trunc-BP-R	MD15G1313200	GGGGACCACTTTGTACAAGAAAGCTGGGTCTAGCCATTAGATTTCTCCACCAC	This work
	MdFLC-BP-F	MD09G1009100	GGGGACAAGTTTGTACAAAAAAGCAGGCTCTATGGGGCGAGGGAAGGTGCAG	This work
	MdFLC-BP-R	MD09G1009100	GGGGACCACTTTGTACAAGAAAGCTGGGTTTAAAACAACTGTAGTATGGTGGCCG	This work
	MdFLC-trunc-BP-R	MD09G1009100	GGGGACCACTTTGTACAAGAAAGCTGGGTCTAAGTGCTCTGCCGATCCGCTTC	This work
	MdFLC-noStop-BP-R	MD09G1009100	GGGGACCACTTTGTACAAGAAAGCTGGGTtAAACAACTGTAGTATGGTGGCCG	Estevan *et al.*, 2020
	GR-BP-R	pBEACON-GR	GGGGACCACTTTGTACAAGAAAGCTGGGTCTAGCATGGCCGTTTTTGAT	Estevan *et al.*, 2020
	SVP-BP-F	AT2G22540	GGGGACAAGTTTGTACAAAAAAGCAGGCTCTATGGCGAGAGAAAAGATTCAGATC	This work
	SVP-BP-R	AT2G22540	GGGGACCACTTTGTACAAGAAAGCTGGGTCTAACCACCATACGGTAAGCCG	This work
	Venus-BP-F	-	GGGGACAAGTTTGTACAAAAAAGCAGGCTCTATGGTGAGCAAGGGCGAG	This work
	Venus-BP-R	-	GGGGACCACTTTGTACAAGAAAGCTGGGTTTACTTGTACAGCTCGTCC	This work
PIPE cloning	pYB187-F	-	GATCTAGGCAGATCACAAGTTTGTAC	This work
	pYB187-R	-	AACGTCGTGACTGGGAAAAC	This work
	pSVP-PIPE-F	-	TCCCAGTCACGACGTTCCGTAAATATCGTCAGTCTCGTA	This work
	pSVP-PIPE-R	-	GTGATCTGCCTAGATCCACAACGAACAAAAAAACCCTAG	This work
RE cloning	MdDAM1-EcoRI-F	MD15G1384500	CCCTACgaattcATGAAGATCAAGATAAAAAAG	This work
	MdDAM1-SalI-R	MD15G1384500	AACCAAgtcgacCGGAAGCCCCAGTTTG	This work
	MdDAM4-EcoRI-F	MD08G1197300	CCCTACgaattcATGGTGAAAAGGATGAATGAGAAG	This work
	MdDAM4-SalI-R	MD08G1197300	AACCAAgtcgacAGGAAGCCCCAGTTTGAGA	This work
	MdSVPa-EcoRI-F	MD01G1038600	CCCTACgaattcATGGCGAGGGAGAAAATTCAG	This work
	MdSVPa-SalI-R	MD01G1038600	AACCAAgtcgacACCAGAGTAAGGCAGCC	This work
	MdFLC-EcoRI-F	MD09G1009100	CCCTACgaattcATGGGGCGAGGGAAGGTGCAG	This work
	MdFLC-SalI-R	MD09G1009100	AACCAAgtcgacAAACAACTGTAGTATGGTGGCCG	This work
Sequencing	pDONR-F	-	GGCAGTTCCCTACTCTCGC	This work
	pDONR-R	-	CATCAGAGATTTTGAGACACGG	This work
	pDEST22-F	-	TGAAGATACCCCACCAAACC	This work
	pDEST22-R	-	CTCTGGCGAAGAAGTCCAAA	This work
	pDEST32-F	-	GACAGCATAGAATAAGTG	This work
	pDEST32-R	-	GTGGCGGCCGTTACTTACT	This work
	pSVP-F-2500	AT2G22540	CGAAACCAAACCAAGCTGAA	This work
	pSVP-F-2000	AT2G22540	TAGGGGGTGAGTGATACGAGC	This work
	pSVP-R-1900	AT2G22540	GCGGAGTCCCTAATGGGC	This work
	pSVP-F-1500	AT2G22540	CTGTTGTTGACGTTGGTGAAGTA	This work
	pSVP-F-1000	AT2G22540	TGGATTCTTACAAACCAAGGC	This work
	pSVP-F-100	AT2G22540	CTCATTCTTGAATCTTGATCCATC	This work
	BlpR-F	pYB187	CTTTATTGCCAAATGTTTGAACG	This work
	BlpR-R	pYB187	CGTTTGGAACTGACAGAACC	This work

New Phytologist

Methods S1 Supporting methods.

Plant material

'Royal Gala' trees grafted on 'M9' rootstocks were distributed in three sampling blocks of four plants each in an orchard located at SudExpé experimental station in Marsillargues, France. Three dormant bud meristems were harvested per plant in eight timepoints from November 11th 2016 to February 27th 2017. Samples were immediately frozen in liquid nitrogen in the field and stored at −80 °C until use. Once a week after January 2017, shoots (axillary branches of around 50 cm) containing 10 buds each were harvested in the field and the dormant stage of the buds was evaluated under forcing conditions (16 h light/8 h dark and 22 °C) using the Tabuenca's test (Tabuenca, 1964).

Gene expression studies

Total RNA was isolated using the Spectrum™ Plant Total RNA kit (Sigma-Aldrich), and DNase-treated using the TURBO DNA-free Kit (Ambion). The SuperScript™ III First-Strand Synthesis System (Thermo Fisher Scientific) was used for cDNA synthesis according to manufacturer's instructions. Real-time PCR was performed using the LightCycler 480 instrument (Roche), and relative expression was calculated using the $2^{(-\Delta\Delta Ct)}$ method as described (Livak & Schmittgen, 2001; Falavigna *et al.*, 2018).

Complementation assay in Arabidopsis

The CDS of *MdDAM1*, *MdDAM2*, *MdDAM4*, *MdDAMb*, *MdSVPa*, *MdSVPb*, *SVP* and *Venus* were independently introduced into a modified *pGreen0229* Gateway vector (*pYB187*, kindly provided by Dr. Youbong Hyun, MPIPZ, Cologne) (Hellens *et al.*, 2000) in which the p*35S* promoter was replaced by the Arabidopsis *SVP* promoter. To this end, overlapping primers were designed to amplify a region of 3 kb upstream of the TSS of *SVP* with complementary borders to the *pYB187* vector (Table **S1**). In parallel, PCR reactions were performed to amplify the *pYB187* plasmids carrying the cloned CDS sequences. Overlapping fragments were assembled by Polymerase Incomplete Primer Extension (Stevenson *et al.*, 2013).

Tobacco co-immunoprecipitation

A modified pEarleyGate301 (Earley *et al.*, 2006) Gateway vector (kindly provided by Dr. Diarmuid O'Maoileidigh, MPIPZ, Cologne), in which the *att* borders, the *HA* and the *OCS* sites were replaced by the Pro*35Sx2*::*NLS-Venus* cassette, and Pro*35S*::*5xMyC-AtHB33* were used as controls. For each combination, two tobacco plants were used, and three leaves per plant were infiltrated. Tobacco plants were incubated at room temperature for 3 days, and leaves were harvested and frozen in liquid nitrogen. Tobacco leaves were ground in liquid nitrogen and resuspended in extraction buffer (50 mM Tris–HCl pH 7.5, 150 mM NaCl, 10% glycerol, 2 mM EDTA pH 8 with HCl, 5 mM DTT, 0.2% Triton, plant protease inhibitor cocktail (Sigma)). 35 ug of total protein was mixed with 2X Laemmli Buffer to be used as input. Protein concentration was normalized to 0.7 mg of total protein, and the supernatant was incubated with the GFP-trap®_A kit (Chromotek) to immunoprecipitate GFP-fused proteins. Beads were washed three times, resuspended in 2X Laemmli Buffer and boiled at 96 °C for 10 min.

Protein production and purification for EMSA and seq-DAP-seq experiments

The full-length CDS sequences of *MdDAM1*, *MdDAM4*, *MdDAMb*, *MdFLC* and *MdSVPa* were amplified without the stop codon and the restriction sites of *EcoRI* and *SalI* were added to their 5' and 3', respectively (Table **S1**). Each gene was amplified and cloned into the double *EcoRI*- and *SalI*-digested *XLp34* and *XLp39* vectors (Lai *et al.*, 2020), fusing the genes to 3xFLAG and 5xMyC, respectively, to avoid epitope-tag interference to the N-terminal MADS DNA-binding domain. In pairs, tagged proteins were simultaneously produced *in vitro* using the TNT® SP6 High-Yield Wheat Germ Protein Expression System (Promega). Protein complexes were purified using anti-FLAG magnetic beads (Merck Millipore), and Western blots were performed as previously described. For EMSA and seq-DAP-seq assays, MdDAM1-, MdDAM4-, or MdFLC-MyC were independently co-produced *in vitro* with MdSVPa-FLAG, and each complex was sequentially purified using anti-FLAG and anti-MyC beads. For the homomeric complexes, only MyC-tagged protein versions were produced and immunoprecipitated using anti-MyC beads. DNA libraries were prepared maintaining the natural DNA methylation state of the samples. Thirteen seq-DAP-seq libraries were generated using apple genomic DNA extracted from Gala endodormant buds,

three replicates for MdDAM1–MdSVPa, MdDAM4–MdSVPa, MdFLC–MdSVPa and MdSVPa–MdSVPa, and the input DNA as a control.

Calli transformation and RNA-seq

In vitro cuttings of apple cultivar Gala were subcultured, and apple leaf transformation was carried out to produce transformed calli as described elsewhere (Estevan *et al.*, 2020). Positive transformed calli were selected in media supplemented with antibiotics and the observation of GFP fluorescence. Six months after leaf transformation, 30 transformed calli were obtained for each construct. Transformed calli were pretreated with 40 µM CHX (Sigma-Aldrich) for 30 min, rinsed with distilled water, and treated with 10 µM DEX (Sigma-Aldrich) or mock (ethanol). After 1 h, samples were rinsed with distilled water, incubated at room temperature for 7 h and then were frozen in liquid nitrogen and stored at −80 °C.

Bioinformatic analyses

Read cleaning and mapping of seq-DAP-seq assays

Raw seq-DAP-seq reads were pre-processed by removing potential sequence adapters with Cutadapt (Martin, 2011) and trimming of low-quality bases ($Q < 15$) at the ends using Trimmomatic (Bolger *et al.*, 2014). All pre-processed reads with final lengths smaller than 50 bases were discarded. Cleaned reads were mapped to the apple double-haploid genome version 1.1 (Daccord *et al.*, 2017) using BWA (Li & Durbin, 2009) with default settings. Raw BWA alignments were filtered by only keeping alignment pairs with mapping quality of at least 20 and by removing secondary alignments.

Peak calling and annotation

Raw seq-DAP-seq peaks were individually called together with a single control sample (input) using the program MACS2 (Zhang *et al.*, 2008) (parameters: -p 0.05 -g 550601767). Individual peak sets were called for all three replicates of the four different complexes. Reproducibility between the replicate peak sets was assessed using the irreproducible discovery rate (IDR) of 0.01 with the framework available from the BioConda channel of the conda package

manager (Dale *et al.*, 2018). The final peak sets were generated by merging overlapping peaks that passed the IDR assessment. Peaks were annotated to apple genes (3 kb up- and 1 kb downstream) using the Bioconductor R package ChIPpeakAnno (Zhu *et al.*, 2010).

Peak position analysis

The preferred location of peaks relative to genes was analyzed using a previously described approach (Romera-Branchat *et al.*, 2020). Briefly, for every peak annotated to a gene, the distance between the peak center to the TSS or TES of the respective gene was calculated depending on whether the peak was located upstream or downstream of it, respectively. For those peaks residing within the gene body, a normalized distance was calculated in relation to the TSS. The normalized distance was obtained using a gene-specific length factor that sets the gene length to 2,000 nucleotides. The observed peak location distribution was compared to that of 1,000 random peak sets consisting of the exact number of peaks with the same length as the observed peak set.

Identification of enriched motifs

De novo motif enrichment analysis was performed using MEME-chip (Ma *et al.*, 2014). For that, the central 100 nucleotides of the peaks were used for motif discovery (parameters: meme-chip -meme-minw 8 -meme-maxw 15 -meme-nmotifs 15 -meme-mod anr -db JASPAR2018_CORE_plants_non-redundant_pfms_meme.txt -centrimo-local -centrimo-ethresh 1), provided with binding sites of plant-specific transcription factors obtained from the JASPAR webpage (http://jaspar.genereg.net). The occurrences of predicted motifs were extracted from the corresponding FIMO output produced by the MEME-ChIP suite. Complementarily, a custom C++ code was written to manually search the peaks for the presence of a previously described degenerated CArG-box motif, MYHWAWWWRGWWW (Mateos *et al.*, 2017; Tilmes *et al.*, 2019). To each complex, we compared the number of peaks associated with genes and containing degenerated CArG-box motifs to the frequency distribution of CArG boxes in 1,000 random sets. To generate each random set, the distance from the start of the peak to the nearest TSS was recorded for each observed peak. Next, a random gene was selected, and a sequence with the

same size and with the same distance from the TSS of a corresponding observed peak was obtained. Finally, the number of peaks with degenerated CArG-box sequences was determined in each random set.

RNA-seq analyses

The RNA-seq reads of the GR DEX-inducible assays were pre-processed as described above (see "Read cleaning and mapping of seq-DAP-seq assays"). Cleaned reads were mapped to the apple genome using TopHat2 (Kim *et al.*, 2013) (parameters: -i 50 -I 10000 -N 5 --read-edit-dist 5 --read-gap-length 2). The apple genome annotation was used for intron hints. The number of reads mapped to each gene was determined using the HTSeq count program (Anders *et al.*, 2015). Differential gene expression analyses were carried out using the DESeq2 R package (Love *et al.*, 2014). The publicly available transcriptome samples PRJNA374502 (dataset A (Moser *et al.*, 2020)) and PRJDB6779 (dataset B (Takeuchi *et al.*, 2018)) were downloaded from the NCBI sequence read archive and quantified using the Salmon program (Patro *et al.*, 2017). Differential gene expression analyses were performed as previously described. For dataset A, pairwise comparisons were performed between dormant samples harvested from October to February (endo- to ecodormancy transition) in comparison to budbreak samples harvested in March. A similar strategy was employed for dataset B, and samples harvested 10, 25, 35 and 65 days after cold treatment were compared to endodormant samples obtained in the field before artificial cold exposure.

Gene clustering

FPKM gene expression values for dataset A (Moser *et al.*, 2020) were obtained using the fpkm function of DESeq2 (Love *et al.*, 2014). The expression values were transformed into the z-scale and clustered using the scale and hclust functions of R (R, 2020), respectively. The dendrograms from the heatmaps generated from the z-transformed expression data were visually inspected and a cutoff value was arbitrarily chosen in order to obtain the gene clusters. The expression data for each cluster was summarized by the eigen gene, which was obtained using the moduleEigengenes function of the R package WGCNA (Langfelder & Horvath, 2008).

GO enrichment tests

A BLAST (Altschul *et al.*, 1997) similarity search (parameters: -F F -e 1e-10 -b 1 -v 1) was performed using the apple proteins as a query against the Arabidopsis TAIR10 proteome (www.arabidopsis.org). The apple proteins/genes inherited the GO term annotation of their best Arabidopsis counterpart.

The 'plant GO slim' subset was obtained from the GO website (http://current.geneontology.org/ontology/subsets/goslim_plant.obo). All enrichment tests using the 'plant GO slim' annotations were performed in MATLAB. By using the geneont class from the MATLAB Bioinformatics toolbox, genes were annotated with a particular GO slim term if one of its originally blast-based GO terms corresponds to or is a descendant of that GO slim category. The GO term enrichment in a gene set was assessed using the hypergeometric test. Raw P-values were adjusted for multiple testing using the Benjamin Hochberg method (Benjamini & Hochberg, 1995). For the enrichment analysis of the seq-DAP-seq targets of the four MADS complexes, the background consisted of all genes in the apple genome with at least one GO slim annotation. The test sets encompassed the target genes of each MADS complex having the specific GO slim terms to be tested. The results were displayed as heatmaps with a grayscale reflecting the P-value. For the enrichment analysis using expression data from the calli assay, the background was determined as the set of genes tested by DESeq2 (in a MADS TF-dependent manner) and assigned to at least one GO slim term. The test sets consisting of GO-annotated DEGs were then separated into up- or downregulated gene sets, and both sets were individually tested for GO slim term enrichment. The results were plotted as a heatmap in which an orange or blue gradient was assigned in the event that the upregulated or the downregulated set, respectively, was enriched ($P \leq 0.05$). In case both sets were enriched, the set with the best P-value was kept.

The GO term enrichment analysis for the gene clusters obtained from dataset A (Moser *et al.*, 2020) was performed using the Cytoscape plugin BiNGO (Maere *et al.*, 2005). For these tests, the background consisted of all GO annotated apple genes. The test sets consisted of the genes within a particular cluster having the GO term being tested.

Supporting References

Altschul SF, Madden TL, Schäffer AA, Zhang J, Zhang Z, Miller W, Lipman DJ. **1997**. Gapped BLAST and PSI-BLAST: a new generation of protein database search programs. *Nucleic Acids Research* **25**: 3389–3402.

Anders S, Pyl PT, Huber W. **2015**. HTSeq--a Python framework to work with high-throughput sequencing data. *Bioinformatics* **31**: 166–169.

Benjamini Y, Hochberg Y. **1995**. Controlling the False Discovery Rate: A Practical and Powerful Approach to Multiple Testing. *Journal of the Royal Statistical Society: Series B (Methodological)* **57**: 289–300.

Bolger AM, Lohse M, Usadel B. **2014**. Trimmomatic: a flexible trimmer for Illumina sequence data. *Bioinformatics* **30**: 2114–2120.

Daccord N, Celton J, Linsmith G, Becker C, Choisne N, Schijlen E, van de Geest H, Bianco L, Micheletti D, Velasco R, *et al.* 2017. High-quality de novo assembly of the apple genome and methylome dynamics of early fruit development. *Nature Genetics* **49**: 1099–1106.

Dale R, Grüning B, Sjödin A, Rowe J, Chapman BA, Tomkins-Tinch CH, Valieris R, Batut B, Caprez A, Cokelaer T, *et al.* 2018. Bioconda: Sustainable and comprehensive software distribution for the life sciences. *Nature Methods* **15**: 475–476.

Earley KW, Haag JR, Pontes O, Opper K, Juehne T, Song K, Pikaard CS. **2006**. Gateway-compatible vectors for plant functional genomics and proteomics. *Plant Journal* **45**: 616–629.

Estevan J, Gómez-Jiménez S, Falavigna V da S, Camuel A, Planel L, Costes E, Andrés F. **2020**. An efficient protocol for functional studies of apple transcription factors using a glucocorticoid receptor fusion system. *Applications in Plant Sciences* **8**: e11396.

Falavigna V da S, Porto DD, Miotto YE, Santos HP dos, Oliveira PRD de, Margis-Pinheiro M, Pasquali G, Revers LF. **2018**. Evolutionary diversification of galactinol synthases in Rosaceae: adaptive roles of galactinol and raffinose during apple bud dormancy. *Journal of Experimental Botany* **69**: 1247–1259.

Hellens RP, Anne Edwards E, Leyland NR, Bean S, Mullineaux PM. **2000**. pGreen: A versatile and flexible binary Ti vector for Agrobacterium-mediated plant transformation. *Plant Molecular Biology* **42**: 819–832.

Kim D, Pertea G, Trapnell C, Pimentel H, Kelley R, Salzberg SL. **2013**. TopHat2: accurate alignment of transcriptomes in the presence of insertions, deletions and gene fusions. *Genome Biology* **14**: R36.

Lai X, Stigliani A, Lucas J, Hugouvieux V, Parcy F, Zubieta C. **2020**. Genome-wide binding of SEPALLATA3 and AGAMOUS complexes determined by sequential DNA-affinity purification sequencing. *Nucleic Acids Research* **48**: 9637–9648.

Langfelder P, Horvath S. **2008**. WGCNA: an R package for weighted correlation network analysis. *BMC Bioinformatics* **9**: 559.

Li H, Durbin R. **2009**. Fast and accurate short read alignment with Burrows-Wheeler transform. *Bioinformatics* **25**: 1754–1760.

Livak KJ, Schmittgen TD. **2001**. Analysis of relative gene expression data using real-time quantitative PCR and the 2^(-ΔΔCT) method. *Methods* **25**: 402–408.

Love MI, Huber W, Anders S. **2014**. Moderated estimation of fold change and dispersion for RNA-seq data with DESeq2. *Genome Biology* **15**: 550.

Ma W, Noble WS, Bailey TL. **2014**. Motif-based analysis of large nucleotide data sets using MEME-ChIP. *Nature Protocols* **9**: 1428–1450.

Maere S, Heymans K, Kuiper M. **2005**. BiNGO: a Cytoscape plugin to assess overrepresentation of Gene Ontology categories in Biological Networks. *Bioinformatics* **21**: 3448–3449.

Martin M. **2011**. Cutadapt removes adapter sequences from high-throughput sequencing reads. *EMBnet.journal* **17**: 10.

Mateos JL, Madrigal P, Tsuda K, Rawat V, Richter R, Romera-Branchat M, Fornara F, Schneeberger K, Krajewski P, Coupland G. **2015**. Combinatorial activities of SHORT VEGETATIVE PHASE and FLOWERING LOCUS C define distinct modes of flowering regulation in Arabidopsis. *Genome Biology* **16**: 31.

Mateos JL, Tilmes V, Madrigal P, Severing E, Richter R, Rijkenberg CWM, Krajewski P, Coupland G. **2017**. Divergence of regulatory networks governed by the orthologous transcription factors FLC and PEP1 in Brassicaceae species. *Proceedings of the National Academy of Sciences* **114**: E11037–E11046.

Moser M, Asquini E, Miolli GV, Weigl K, Hanke M, Flachowsky H, Si-Ammour A. **2020**. The MADS-Box Gene MdDAM1 Controls Growth Cessation and Bud Dormancy in Apple. *Frontiers in Plant Science* **11**: 1003.

Patro R, Duggal G, Love MI, Irizarry RA, Kingsford C. **2017**. Salmon provides fast and bias-aware quantification of transcript expression. *Nature Methods* **14**: 417–419.

Perini P, Pasquali G, Margis-Pinheiro M, de Oliviera PRD, Revers LF. **2014**. Reference genes for transcriptional analysis of flowering and fruit ripening stages in apple (Malus × domestica Borkh.). *Molecular Breeding* **34**: 829–842.

Porto DD, Bruneau M, Perini P, Anzanello R, Renou J-P, Santos HP dos, Fialho FB, Revers LF. **2015**. Transcription profiling of the chilling requirement for bud break in apples: a putative role for FLC-like genes. *Journal of Experimental Botany* **66**: 2659–2672.

Porto DD, da Silveira Falavigna V, Arenhart RA, Perini P, Buffon V, Anzanello R, dos Santos HP, Fialho FB, de Oliveira PRD, Revers LF. **2016**. Structural genomics and transcriptional characterization of the Dormancy-Associated MADS-box genes during bud dormancy progression in apple. *Tree Genetics & Genomes* **12**: 46.

R CT. **2020**. R: A Language and Environment for Statistical Computing.

Romera-Branchat M, Severing E, Pocard C, Ohr H, Vincent C, Née G, Martinez-Gallegos R, Jang S, Andrés F, Madrigal P, *et al.* **2020**. Functional Divergence of the Arabidopsis Florigen-Interacting bZIP Transcription Factors FD and FDP. *Cell Reports* **31**: 107717.

Stevenson J, Krycer JR, Phan L, Brown AJ. **2013**. A practical comparison of ligation-independent cloning techniques. *PLoS ONE* **8**: 8–14.

Tabuenca MC. **1964**. Chilling requirements of apricot, peach and pear varieties. *Aula Dei*: 113–132.

Takeuchi T, Matsushita MC, Nishiyama S, Yamane H, Banno K, Tao R. **2018**. RNA-sequencing Analysis Identifies Genes Associated with Chilling-mediated Endodormancy Release in Apple. *Journal of the American Society for Horticultural Science* **143**: 194–206.

Tilmes V, Mateos JL, Madrid E, Vincent C, Severing E, Carrera E, López-Díaz I, Coupland G. **2019**. Gibberellins Act Downstream of Arabis PERPETUAL FLOWERING1 to Accelerate Floral Induction

during Vernalization. *Plant Physiology* **180**: 1549–1563.
Wu R, Tomes S, Karunairetnam S, Tustin SD, Hellens RP, Allan AC, Macknight RC, Varkonyi-Gasic E. **2017**. SVP-like MADS Box Genes Control Dormancy and Budbreak in Apple. *Frontiers in Plant Science* **8**: 477.
Zhang Y, Liu T, Meyer CA, Eeckhoute J, Johnson DS, Bernstein BE, Nussbaum C, Myers RM, Brown M, Li W, *et al.* 2008. Model-based Analysis of ChIP-Seq (MACS). *Genome Biology* **9**: R137.
Zhu LJ, Gazin C, Lawson ND, Pagès H, Lin SM, Lapointe DS, Green MR. **2010**. ChIPpeakAnno: a Bioconductor package to annotate ChIP-seq and ChIP-chip data. *BMC Bioinformatics* **11**: 237.
