# Supplementary material for: Unraveling the role of MADS transcription factor complexes in apple tree dormancy
Source: New Phytol. 2021 Sep 23;232(5):2071–88. doi: 10.1111/nph.17710 (PMC9292984; doi:10.1111/nph.17710)

New Phytologist

***New Phytologist* Supporting Information**

Article title: Unraveling the role of MADS transcription factor complexes in apple tree dormancy

Authors: Vítor da Silveira Falavigna, Edouard Severing, Xuelei Lai, Joan Estevan, Isabelle Farrera, Véronique Hugouvieux, Luís Fernando Revers, Chloe Zubieta, George Coupland, Evelyne Costes, Fernando Andrés

Article acceptance date: 19 August 2021

Data S5. Heatmap summarizing the gene expression during dormancy of the high-confidence targets of MdDAM1–MdSVPa, MdDAM4–MdSVPa and MdFLC–MdSVPa complexes. The Z-score expression values corresponding to the colors in the heatmap are indicated by the colormap. The histogram in the colormap indicates the frequencies of each color in the heatmap.

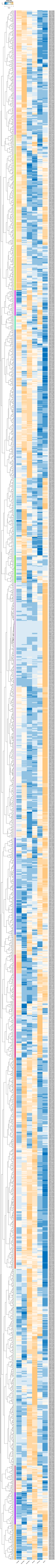

Supplement: Supplementary file 5 — Dataset S5 Heatmap summarizing the gene expression during dormancy of the high‐confidence targets of the MdDAM1–MdSVPa, MdDAM4–MdSVPa and MdFLC–MdSVPa complexes. [file NPH-232-2071-s011.pdf]
